# Electron Tomography and Simulation of Baculovirus Actin Comet Tails Support a Tethered Filament Model of Pathogen Propulsion

**DOI:** 10.1371/journal.pbio.1001765

**Published:** 2014-01-14

**Authors:** Jan Mueller, Julia Pfanzelter, Christoph Winkler, Akihiro Narita, Christophe Le Clainche, Maria Nemethova, Marie-France Carlier, Yuichiro Maeda, Matthew D. Welch, Taro Ohkawa, Christian Schmeiser, Guenter P. Resch, J. Victor Small

**Affiliations:** 1 Institute of Molecular Biotechnology, Austrian Academy of Sciences, Vienna, Austria; 2 RICAM, Austrian Academy of Sciences, Vienna, Austria; 3 Faculty of Mathematics, University of Vienna, Austria; 4 Nagoya University, Graduate School of Sciences, Structural Biology Research Center and Division of Biological Sciences, Nagoya, Japan; 5 Nagoya University JST PRESTO, 4-1-8 Honcho Kawaguchi, Saitama, Japan; 6 Laboratoire d'Enzymologie et Biochimie Structurales, Centre National de la Recherche Scientifique, Gif-sur-Yvette, France; 7 Department of Molecular and Cell Biology, University of California, Berkeley, California, United States of America; 8 Campus Science Support Facilities GmbH, Vienna, Austria; Princeton University, United States of America

## Abstract

Several pathogens induce propulsive actin comet tails in cells they invade to disseminate their infection. They achieve this by recruiting factors for actin nucleation, the Arp2/3 complex, and polymerization regulators from the host cytoplasm. Owing to limited information on the structural organization of actin comets and in particular the spatial arrangement of filaments engaged in propulsion, the underlying mechanism of pathogen movement is currently speculative and controversial. Using electron tomography we have resolved the three-dimensional architecture of actin comet tails propelling baculovirus, the smallest pathogen yet known to hijack the actin motile machinery. Comet tail geometry was also mimicked in mixtures of virus capsids with purified actin and a minimal inventory of actin regulators. We demonstrate that propulsion is based on the assembly of a fishbone-like array of actin filaments organized in subsets linked by branch junctions, with an average of four filaments pushing the virus at any one time. Using an energy-minimizing function we have simulated the structure of actin comet tails as well as the tracks adopted by baculovirus in infected cells *in vivo*. The results from the simulations rule out gel squeezing models of propulsion and support those in which actin filaments are continuously tethered during branch nucleation and polymerization. Since *Listeria monocytogenes*, *Shigella flexneri*, and Vaccinia virus among other pathogens use the same common toolbox of components as baculovirus to move, we suggest they share the same principles of actin organization and mode of propulsion.

## Abstract 2

Electron tomography reveals the structural organization of actin comet tails generated by a baculovirus, providing an understanding of how this pathogen hijacks host machinery to propel itself between cells.

## Author Summary

Several bacteria and viruses hijack the motile machinery of cells they invade to generate networks of actin filaments (comet tails) to propel themselves from one cell to another. A proper understanding of the mechanism of propulsion has so far been hampered by a lack of information about the structure of the machinery. Using electron tomography we present here the three-dimensional structure of actin comet tails propelling a baculovirus, the smallest pathogen known to recruit the actin nano-machinery. We show that baculovirus is propelled by a fishbone-like array of actin filaments constructed from subsets linked by branch junctions, with an average of four filaments pushing the virus by their fast polymerizing ends at any one time. Using a stochastic mathematical model we have simulated comet tail organization as well as the tracks adopted by baculovirus inside cells. The simulations support a model of baculovirus propulsion in which the actin filaments are continuously tethered to the virus surface as they grow, branch, and push. Since larger pathogens like Listeria, Shigella, and Vaccinia virus generate comet tails exhibiting the same general morphology and components as those of baculovirus, the basic mechanism of their propulsion is likely a scaled up version of the one described here.

## Introduction

The seminal finding of Tilney and Portnoy [1] that *Listeria monocytogenes* exploits the actin cytoskeleton of infected cells to invade neighboring cells opened a new chapter in motile processes based on actin. Major progress in understanding how *L. monocytogenes* uses actin to move came from the identification of the Arp2/3 complex [2] as the downstream target promoting actin polymerization [3] and from the subsequent elucidation of the minimal protein cocktail required for propulsion *in vitro* [4]. Essential in the *in vitro* motility mix was actin, the Arp2/3 complex, ADF/cofilin, an actin capping protein and an activator of the Arp2/3 complex, ActA on *L. monocytogenes*, or N-WASP on plastic beads [4],[5]. Subsequent studies have revealed a growing list of bacterial and viral pathogens that exploit the actin-based motile machinery of infected cells by mimicking or recruiting N-WASP to activate the Arp2/3 complex [6],[7] or other actin nucleators [8]. To gain more insight into the mechanism of propulsion, several efforts have been directed at establishing the structure of the actin comet tail induced by pathogens. Electron microscopy of plastic-embedded, negatively stained *L. monocytogenes* comet tails, or critical point dried tails on ActA-coated beads [1],[9]–[11] showed actin filaments more or less randomly oriented, but the high density of filaments precluded definition of their spatial organization by conventional 2D imaging.

Using electron tomography we recently showed that lamellipodia networks comprise subsets of actin filaments linked by branch junctions structurally homologous to those formed from the Arp2/3 complex and actin *in vitro* [12]. With electron tomography practical limits are set by the thickness of the sample and *L. monocytogenes* are more than twice as thick as lamellipodia, making them less suitable for structural analysis. The timely finding that a baculovirus species, *Autographa californica* multiple nucleopolyhedrovirus, just 50 nm in diameter, moves on an actin comet tail in infected cells and bears a minor capsid protein p78/83 that directly activates the Arp2/3 complex [13]–[15] offered an ideal object for resolving comet architecture. In the present report we provide the first structure, to our knowledge, of an actin comet tail driving a pathogen and use this new information to re-evaluate alternative models (reviewed in [16],[17]) of pathogen propulsion by actin.

## Results

### Baculoviruses Generate Actin Comets in Vertebrate Cells

**Figure 1:**
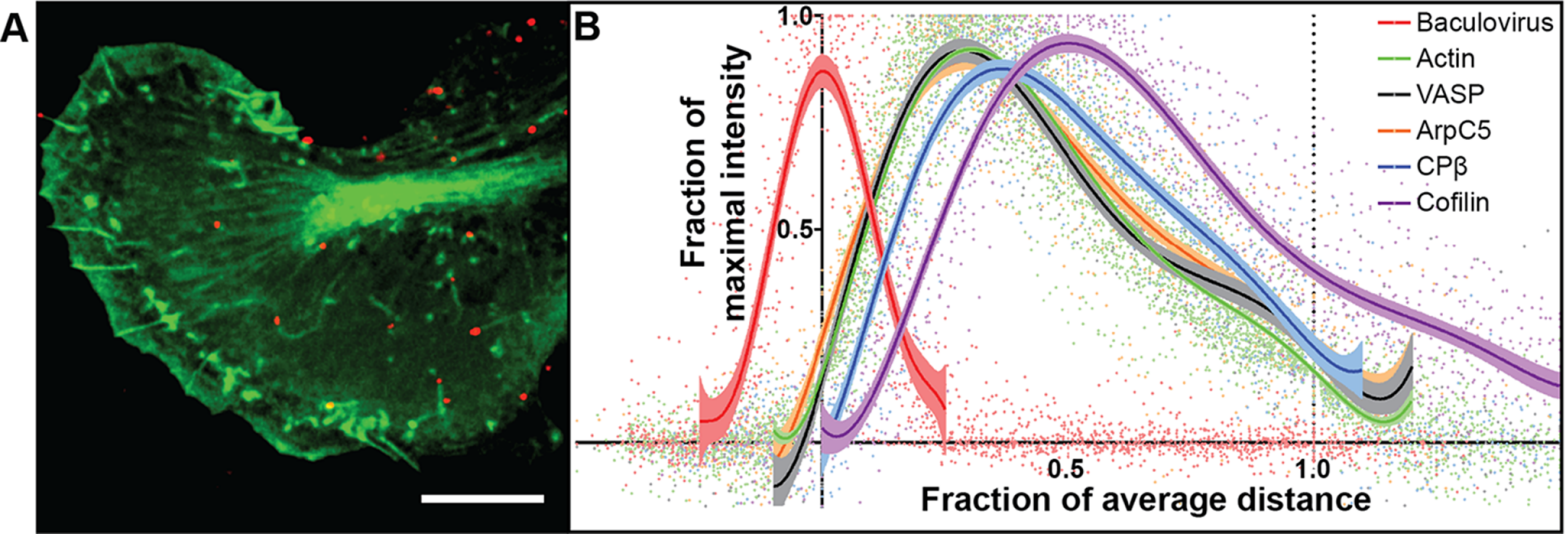
Live cell imaging of baculovirus actin comet tails. (A) Overview of GFP-actin expressing B16 melanoma cell that was infected with baculoviruses tagged with mCherry. (B) Relative distribution of actin and associated proteins along baculovirus comet tails. Tail length averaged 3.8±1.8 µm (s.d., *n* = 111 individual tails) and was normalized to 1.0. Curves were fitted using a centered sixth order polynomial function. Shown are 95% confidence bands for each dataset. For details, see Figure S1. Bar, 10 µm.

Lepidopteran cells, the natural host of baculovirus, proved too thick for analysis by electron tomography. However, we found that baculovirus was readily taken up by thinner vertebrate cells of mouse (B16 melanoma, NIH 3T3 fibroblasts), fish (CAR fibroblasts), and human (HeLa) origin. Figure 1A and Movie S1 show a B16 melanoma cell expressing GFP-actin that was infected with baculovirus tagged with mCherry [14]. The actin comet tails formed were indistinguishable from those seen in the native host cells, moved with a velocity of up to 50 µm/min, and contained typical tail components (Figures 1B and S1) [11]. From intensity measurements of tails in cells transfected with different combinations of GFP/mCherry-tagged proteins, VASP and ArpC5 co-localized with actin and capping protein β and cofilin trailed behind the actin label (Figure 1B and Figure S1).

### Electron Tomography of Intracellular Comet Tails Induced by Baculovirus

By applying cryo-electron tomography to intact cells and cytoskeletons, we previously showed that actin networks in lamellipodia are preserved using a cytoskeleton preparation procedure involving simultaneous extraction with Triton X-100 and fixation in glutaraldehyde [18]. This procedure leads to a marked improvement in filament contrast and resolution as compared to un-extracted cells. Using the same routine with baculovirus infected cells, we could show that the fluorescence intensity profile and length of actin comet tails were retained by this extraction/fixation method (Figure S2).

**Figure 2:**
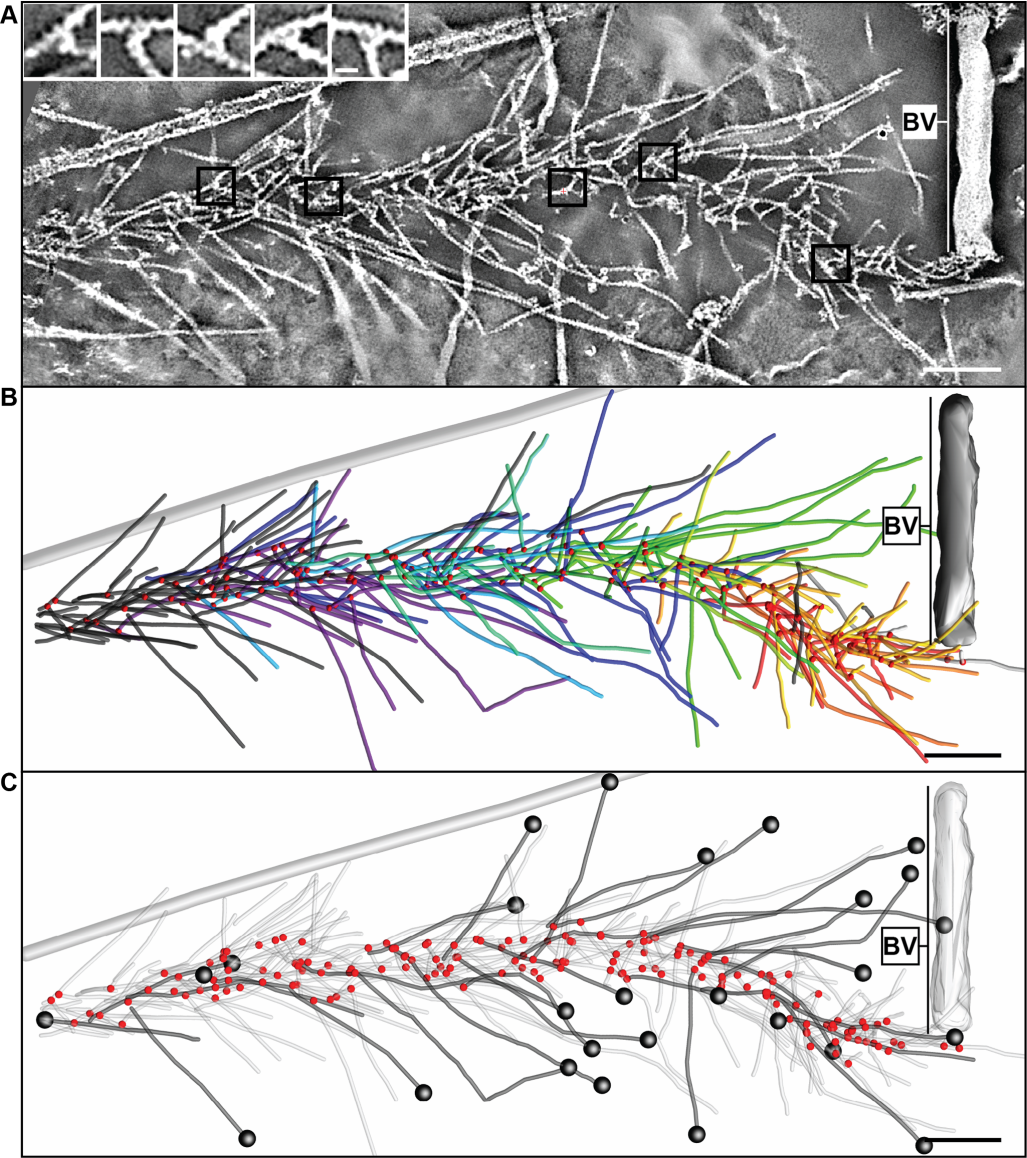
Electron tomography of negatively stained baculovirus actin comet tail *in vivo*. (A) Negatively stained comet tail in a cytoskeleton of a fish fibroblast. Image shows a 14.5 nm section of the tomogram, with the virus particle on the right (BV). Insets show details of branch junctions from the squares in the overview image. (B) Projection of 3D model derived from the tomogram in (A) showing the branch points as red dots. Actin filaments are marked as lines of different colors, with each color depicting filaments linked into a subset by branch junctions. Grey tube corresponds to a microtubule. (C) Projection of 3D model highlighting filaments subjected to polarity analysis in black and the branch points in red. Black spots mark the plus ends of the filaments. See also Figure S3 and Movie S2. Bars (A–C), 100 nm; inset, 10 nm.

**Figure 3:**
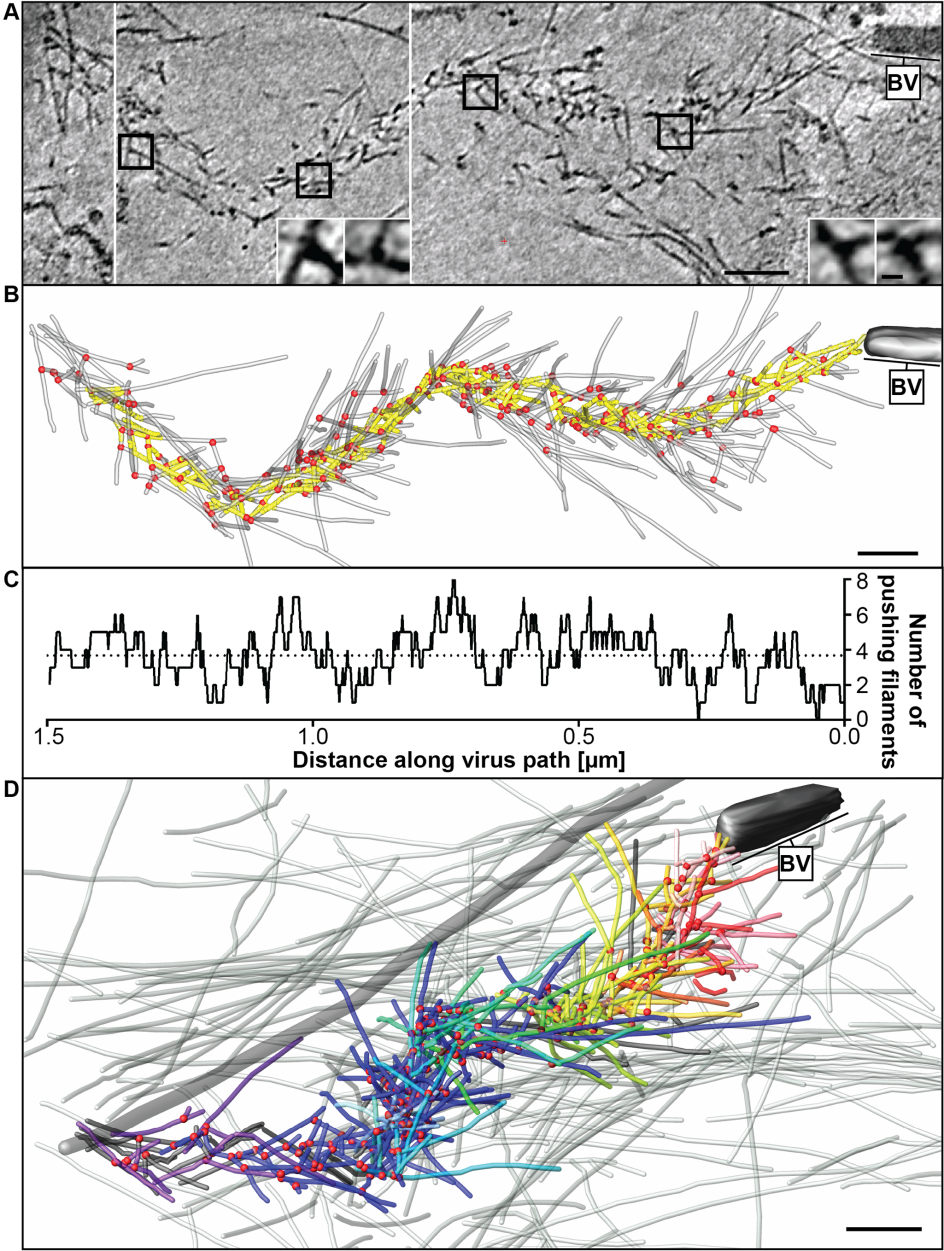
Cryo-electron tomography of a baculovirus actin comet tail *in vivo*. (A) Cryo-electron tomogram of a baculovirus comet tail in a B16 melanoma cell. Image shows 19 nm sections of the tomogram. Since the virus tail was not in one plane in the ice layer, the tomogram is shown in three images, separated by white lines, taken at different z-levels. Insets show details of branch junctions from the squares in the overview image. (B) Projection of 3D model derived from the tomogram in (A) showing the branch points as red dots and actin filaments as translucent lines. Yellow region indicates the core of the tail previously traversed by the cross-section of the virus, used for deriving (C). (C) Plot of the number of filaments transecting the core region in (B), taken as the number involved in pushing. (D) Projection from the rear of the complete comet tail model with actin filaments of the host cytoskeleton (translucent) as well as one microtubule (grey tube) superimposed. See also Movie S3. Bars (A, B), 100 nm; inset, 10 nm.

The ultrastructural organization of baculovirus actin comet tails was resolved in cytoskeletons of infected cells using two complementary approaches involving embedment either in negative stain (Figure 2) or in vitreous ice (Figure 3). A section of an electron tomogram of a negatively stained baculovirus comet tail in the cytoplasm of a fish fibroblast cytoskeleton and the model derived by filament tracking are shown in Figure 2 (see also Movie S2). As shown, the comet tail filaments form a fishbone-like array generated through the frequent formation of branch junctions (highlighted in the insets in Figure 2A) with an average angle of 75.2±8.0° (s.d., *n* = 652). From tracking of filaments in the comet tail network, they appeared to be organized into subsets (averaging 14.2±8.3 filaments per subset, s.d., *n* = 34) highlighted in Figure 2B in different colors, each linked by a separate group of branch junctions (red spots in Figure 2B,C). Cross-correlation analysis [19],[20] of the negatively stained comet tail filaments with reference models of the actin helix (Figure S3) showed that the fast polymerizing, plus ends were directed forwards. The plus ends of the filaments analyzed are highlighted by black spheres in Figure 2C.

The filament array belonging to the actin comets was mainly separated in Z from the actin filaments of the cytoplasm (see additionally Figures S4 and S5), which were typically longer and randomly oriented. Intermingling of the two filament populations also occurred, but it was relatively straightforward to assign filaments to the comets according to additional criteria: 1, termination within the comet core; and 2, extension of filaments from the core towards the virus.

To circumvent the partial collapse of comet tails in negatively stained preparations, we analyzed them also by cryo-electron tomography. Optimal contrast for filament tracking in actin comets was obtained by imaging them in cytoskeletons of infected cells within cytoplasmic regions that lay over holes in the supporting film (Figure 3 and Movie S3). Branch junctions with the typical angle were readily identified (insets, Figure 3A and Movie S4) with the comet tail filaments oriented at an average angle of 50.9±23.7° (s.d., *n* = 485) to the core axis. The number of filaments transecting a core region of the comet tail corresponding to the cross-section of the virus (highlighted in yellow in Figure 3B) at different axial positions is shown in Figure 3C. These data indicated an average number of filaments involved in pushing of 3.9±1.4 (s.d., *n* = 252, range 1–8). Taken together, the data from three negative stain and three cryo-electron tomograms gave an average filament length of 121.1±99.9 nm (s.d., *n* = 961) (cryo 107.8±89.8 nm, s.d., *n* = 477, negative stain 134.2±107.3 nm, s.d., *n* = 482), average branch angles of 75.2±8.0° (s.d., *n* = 652) (cryo 74.7±8.7°, s.d., *n* = 331, negative stain 75.8±7.3°, s.d., *n* = 321), and an average interbranch spacing of 37.7 nm±30.1 nm (s.d., *n* = 290) (cryo 37.1 nm±32.8 nm, s.d., *n* = 154, negative stain 38.4 nm±26.8 nm, s.d., *n* = 136). Figure 3D shows a projection of the 3D model of the comet tail together with (in translucent grey) the endogenous actin filaments of the cytoskeleton that were mainly located above and below the comet (see also Movie S3 and Figure S6).

### Architecture of Baculovirus Actin Comets Formed *in Vitro*

**Figure 4:**
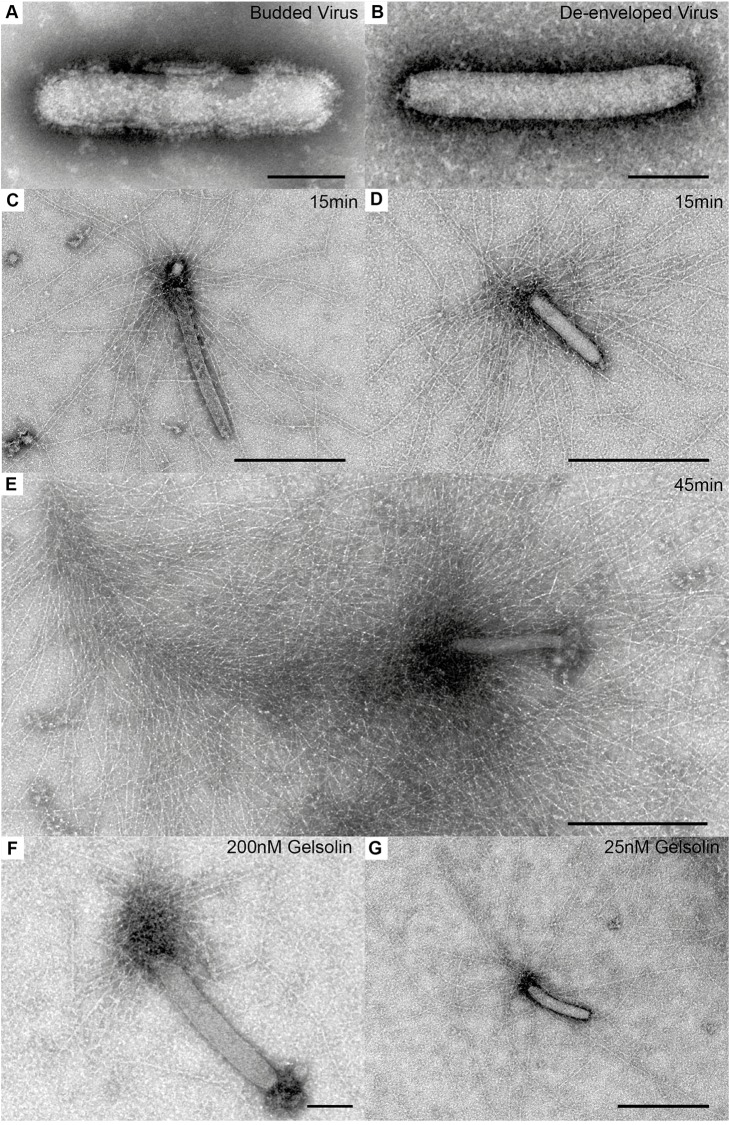
*In vitro* assembled baculovirus comet tails. Images of viruses and actin tails obtained in *in vitro* assays after negative staining and conventional transmission EM. (A) Budded baculovirus in the infected cell supernatant. (B) De-enveloped virus obtained after detergent treatment of the budded virus. (C–E) Actin comet tails formed on baculovirus *in vitro* in the motility cocktail after the incubation times indicated. (F, G) Effect of varying gelsolin concentration on the length of the comet tail filaments. Bars (A, B, F), 100 nm; (C, D, E, G), 500 nm.

**Figure 5:**
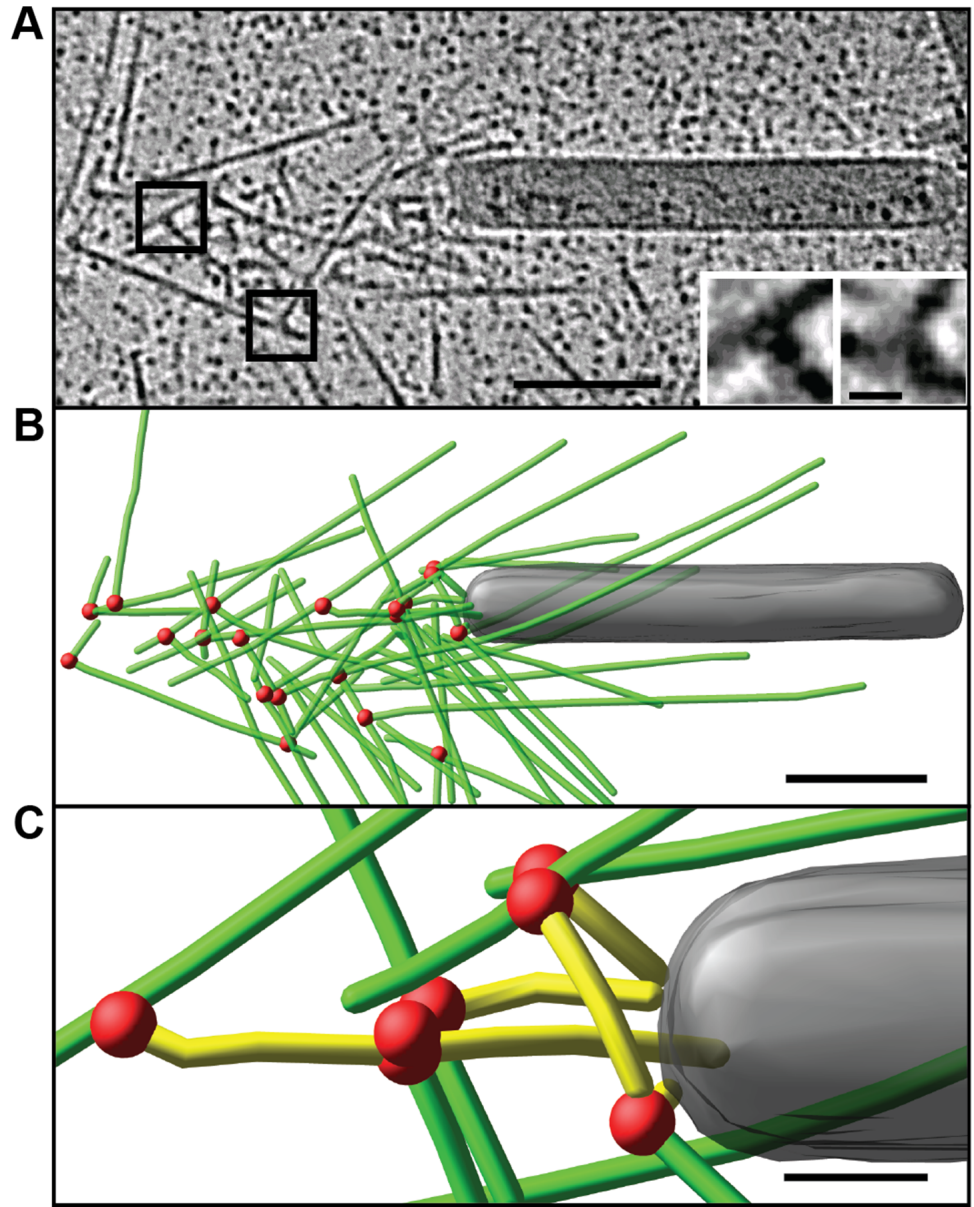
Cryo-electron tomography of baculovirus actin comet tail *in vitro*. (A) Cryo-electron tomogram section (11 nm) of a comet tail formed on a purified, de-enveloped baculovirus *in vitro* in a motility cocktail containing actin, Arp2/3 complex, gelsolin, cofilin, and VASP. Insets show details of branch junctions from the squares in the overview image. (B) Model derived from tomogram, showing actin filaments in green and branch junctions in red. (C) Close-up of the model highlighting the filaments abutting the rear of the virus in yellow. See also Movie S5. Bars (A, B), 100 nm; (C) 25 nm; inset, 10 nm.

By exposing the p78/83 capsid protein of budded baculoviruses from cell supernatants using detergent (Figure 4A,B) we could reconstitute comet tail formation *in vitro* ([4], Figures 4 and 5). In the motility cocktail (actin, Arp2/3 complex, gelsolin, ADF/cofilin, and profilin) filament length was influenced most strongly by variations in gelsolin concentration (Figure 4F,G). Addition of VASP to the protein cocktail substantially increased the frequency of particles bearing actin comets. In all cases actin filaments were nucleated at one end of the virus particle and with appropriate concentrations of cofactors, the *in vitro* comet tails resembled closely those observed *in vivo*. Cryo-electron tomography was used to determine the three-dimensional organization of the *in vitro* comet tails (Figure 5 and Movie S5). As for the *in vivo* comet tails, we observed a fishbone-like array of filaments linked by branch junctions (inset and red spheres, Figure 5) with four to five filaments in close contact with the rear of the virus (Figure 5C). Cross-correlation analysis [19] of individual filaments in the cryo-tomograms (Figure S7) showed that the plus ends were oriented towards the virus, consistent with the analysis of the *in situ* comet tails embedded in a negative stain.

### Mathematical Model of Baculovirus Propulsion

**Figure 6:**
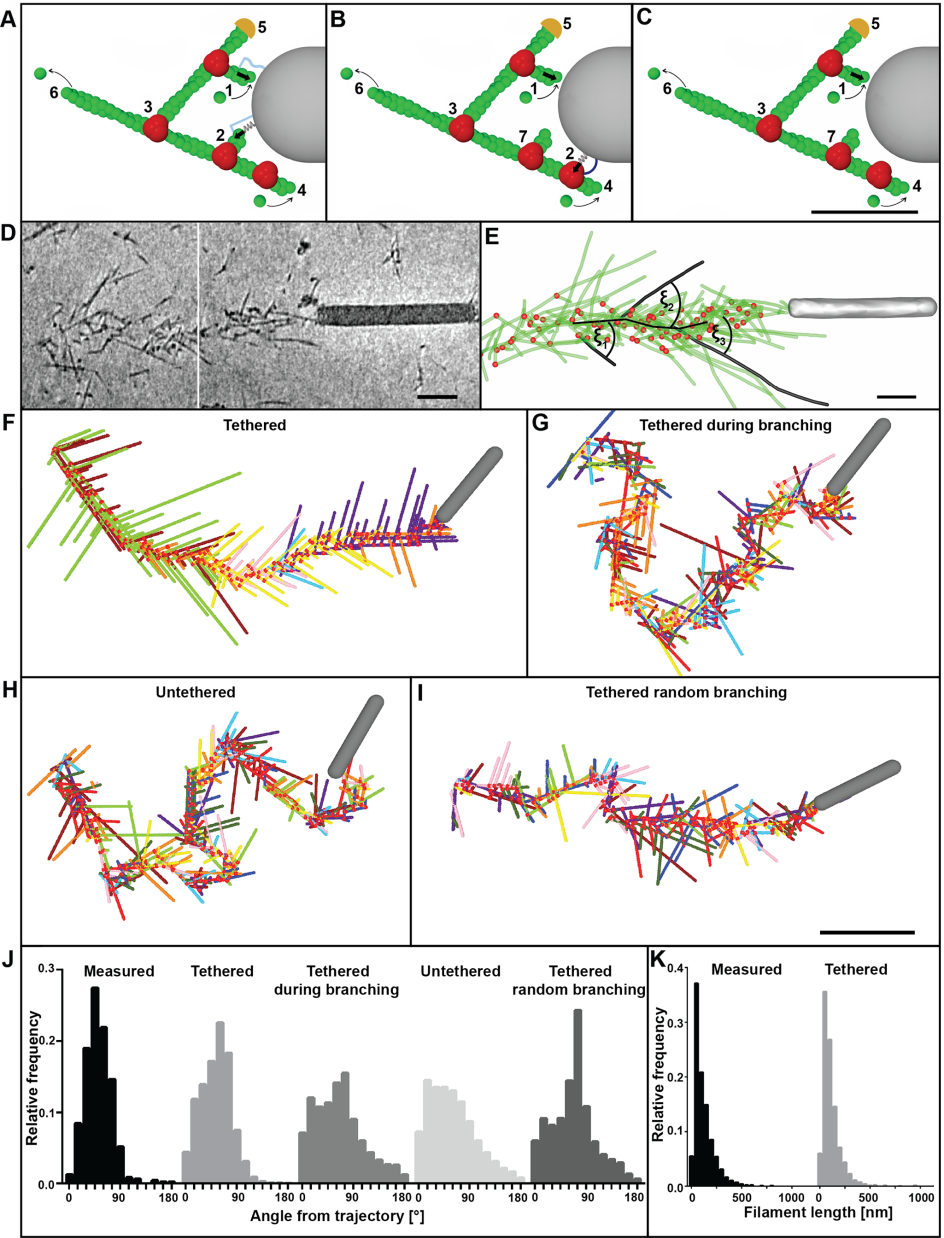
Mathematical simulation of comet tail architecture. (A–C) Schematic representation of the three different models considered. (A). Tethered model. (B) Tethered during branching model. (C) Untethered model. Actin filaments are in green, branchpoints in red, and the virus is depicted in grey. Polymerizing filaments can push (1). The virus surface is continuously tethered to the barbed ends (A, 2) or to the Arp2/3 complex during branching (B, 2) or not at all (C). Tethering is modeled by a spring connection. Branches (3) are initiated by Arp2/3 complex (red) recruited to the plus ends of actin filaments at the virus surface. Elongation of filaments that can no longer push (4) continues until they are capped (5). Filaments at the rear of the tail become de-branched and depolymerize from their minus ends (6). In (B) and (C) filaments lagging behind are not tethered (7). (D) Baculovirus actin comet tail in a B16 melanoma cell observed in vitreous ice, shown in two z-sextons, 18 nm thick. (E) Model derived from tomogram in (D) showing branch points in red and actin filaments in green. The angles of filaments to the core axis are shown for three examples ξ_1_–ξ_3_. (F–I) Simulated comet tails for the different model scenarios: (F) tethered actin filaments; (G) filaments tethered during branching; (H) untethered filaments; (I) tethered filaments but with branching towards and away from the virus surface. Filaments in different colors belong to different subsets. (J) Histograms of angles of filaments to the core trajectory from three cryo-tomograms (*n* = 485) compared to the model simulations with tethered filaments (*n* = 49,321), tethered filaments only during branching (*n* = 58,895), untethered filaments (*n* = 51,168), and filaments with random branching (*n* = 155,556). Measured versus tethered n.s. (*p* = 0.8270); measured versus tethered during branching **** (*p*<0.0001); measured versus untethered **** (*p*<0.0001); Measured versus tethered random branching **** (*p*<0.0001); tethered versus tethered during branching **** (*p*<0.0001); tethered versus untethered **** (*p*<0.0001); tethered versus tethered random branching **** (*p*<0.0001). Data nonparametric, by Kruskal-Wallis test. (K) Histogram of filament lengths for the experimental data (measured) and the “tethered” simulation compared. Bars (A–C), 50 nm, (D, E) 100 nm, (F–I) 300 nm.

**Figure 7:**
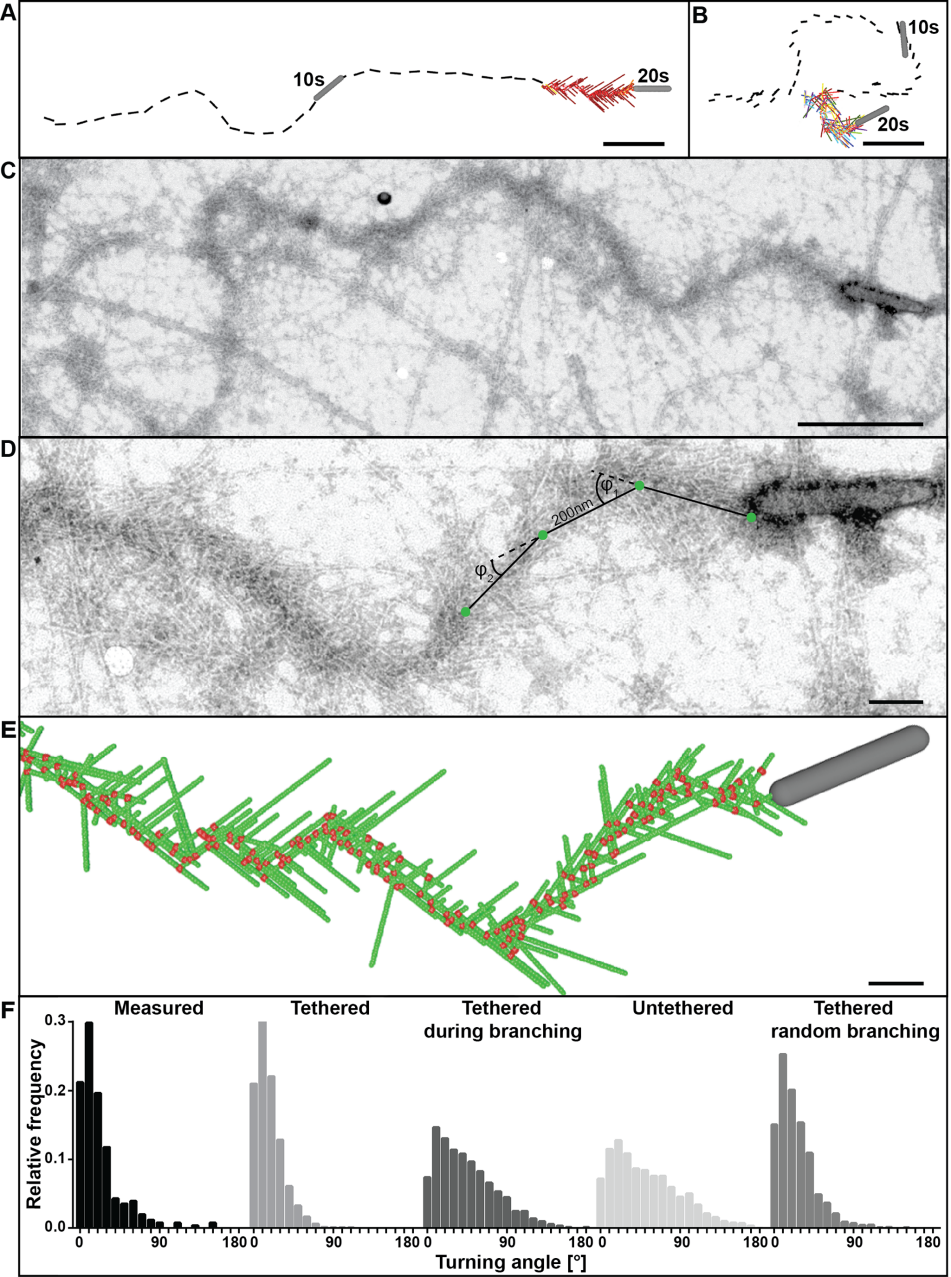
Mathematical simulation of comet tail tracks. (A, B) Simulations of comet tail tracks obtained by assuming that the actin filaments are tethered (A) or untethered (B) to the virus surface. (C, D) Example of a comet tail track observed by conventional negative stain electron microscopy in a CAR fibroblast. The superimposed lines, 200 nm in length, are examples of those used for quantification of angular deviations (angles marked as φ_1_ and φ_2_). (E) Simulated model matching the example in (D). Actin filaments are in green and branch junctions in red. (Actin filament subsets are not shown.) (F) Histograms of angular deviations of comet tail tracks in electron micrographs as in (D) compared to simulations with filaments tethered, untethered, tethered during branching, and tethered with random branching. Measured versus tethered n.s. (*p*>0.9999); measured versus tethered during branching **** (*p*<0.0001); measured versus untethered **** (*p*<0.0001); measured versus tethered random branching n.s. (*p* = 0.0905); tethered versus tethered during branching **** (*p*<0.0001); tethered versus untethered **** (*p*<0.0001); tethered versus tethered random branching **** (*p*<0.0001). Data nonparametric, by Kruskal-Wallis test. Bars, (A–C) 500 nm, (D, E) 100 nm.

Using a quasi-static approach in a two-dimensional model (see Text S1) we sought to mimic the intracellular movement of baculovirus and the structural features of the comet tails. We assumed several forces acting on the virus: Cytoplasmic friction, Brownian forces, and the forces exerted by the actin filaments. In the model, actin filaments are simulated as stiff, immobile rods, which can push the virus due to polymerization. We initially considered three variations of the model (Figure 6A–C). In the “tethered” case (Figure 6A) actin filaments proximal to the virus surface are continuously tethered, both during pushing and when they lag behind. When lagging behind they exert a pulling force described by a spring connection (Figure 6A, 2). In the “tethered during branching” case (Figure 6B) tethering to the virus occurs only during branching events (Figure 6B, 2) and is described by a spring connection to the Arp2/3 complex. In the “untethered” case (Figure 6C) no tethering takes place between the filament plus ends or the branch points with the virus surface. In the latter two cases, the untethered, lagging filaments (Figures 6B,C and 7) may convert into pushing or capped filaments. The primary difference between the three cases is then the degree of pulling forces exerted on the virus by the filament network. In each case, Arp2/3-dependent branches (marked in red in Figure 6A–C) are induced by the p78/83 nucleation promoting factor at the rear of the pathogen. Filaments that move laterally off the rear of the virus continue to polymerize (Figure 6A–C, 4) until they are capped (Figure 6A–C, 5), and those at the rear of the tail de-polymerize from their minus ends (Figure 6A–C, 6).

To compare the electron microscope data with the simulations, we quantified (1) the angles that filaments subtended with the axis of the comet tails (Figure 6D,E), (2) the length distribution of the filaments (Figure 6K), and (3) the number of filaments joined together in subsets (Figure 2B). By an appropriate choice of input parameters (Table S1) we could mimic the measured parameters only for the “tethered” case (Figure 6F,J,K; summarized in Tables S2 and S3). In the “tethered” situation, the distribution of angles subtended to the trajectory (52.6±26.8°, s.d., *n* = 49,321) compared closely to the value obtained from three cryo-tomograms (50.9±23.7°, s.d., *n* = 485), whereas the “tethered during branching” and “untethered” models produced tails with filaments emanating at significantly higher angles (64.8±42.6°, s.d., *n* = 58,895, and 59.7±41.1°, s.d., *n* = 51,168, respectively; Figure 6F,G,H,J). Pushing the virus along a straight path tends to be dynamically unstable due to its elongated shape [21]. To obtain in the model a persistent and relatively straight movement, an average of 3–4 filaments evenly distributed over the rear of the virus were required at any one time. Due to statistical variation, this condition was not satisfied by a single subset of filaments. Instead, regions on the virus rear, where filaments became sparse, required the engagement of new mother filaments, through *de novo* nucleation or recruitment from the cytoplasm that then initiated new filament subsets. In the “tethered” case, the simulations generated an average of 11 filaments per subset, comparable to the value determined from the tomograms, whereas for the “untethered” case a new mother filament was required after every 2–3 branching events, leading to an average of 3 filaments per subset.

The straightness of tracks adopted by the virus in cells was compared to the simulations by measuring the angular divergence of 200 nm segments along the comet tail axis (Figure 7). Experimental data were derived from 19 comet tails imaged by conventional 2D electron microscopy (Figure 7C,D). Again, the range of observed turning angles fitted best the values obtained with the “tethered” case simulations (Figure 7F). With simulations run for extended periods of time (at least 30 min of real time each), tracking in the “tethered” model generally mimicked the undulating course of baculovirus observed by live cell imaging and electron microscopy (Figure 7A,F and Movies S1 and S6), whereas tracks were highly tortuous in the “untethered” simulations (Figure 7B,F and Movie S6) as also previously reported for simulated trajectories of *L. monocytogenes* [22]. Values of the angular divergence of trajectories from tails acquired by transmission electron microscopy (22.0±24.4°, s.d., *n* = 255 angles in 19 individual tails) and “tethered” simulations (17.4±14.8°, s.d., *n* = 1,449) were significantly smaller than those from “untethered” simulations (51.2±38.0°, s.d., *n* = 2,681) and those from simulations for the case of “tethered during branching” (44.4±33.2°, s.d., *n* = 2,337) (Figure 7F, Table S3).

Although the electron tomography data indicated branching only towards the virus surface, we also considered a “tethered” model in which branching could also occur away from the virus, leading to a subpopulation of filaments unable to engage in pushing. Surprisingly, simulations of this “tethered random branching” case produced relatively straight tracks (Figure 6I) with normal deviations (24.5±20.9, s.d., *n* = 1,430, Figure 7F) but failed to reproduce the typical fishbone-like array of actin filaments. This was reflected in significantly higher filament to trajectory angles as well as in a 40% decrease in the number of filaments per subset. In addition the number of productive, pushing filaments was significantly less (3.47±1.27, s.d., *n* = 3,185,000 compared to 4.10±1.25, s.d., *n* = 1,965,000) despite an increase of 20% in the number of filaments per µm tail length.

## Discussion

The resolution achievable by electron tomography is dependent on a number of factors, not least the thickness of the specimen. Baculovirus is at least 20-fold smaller than *L. monocytogenes*, with a diameter only six times the thickness of an actin filament, making it an ideal object for electron tomography. For cryo-electron tomography, we found that filament tracking and polarity analysis was only possible in energy-filtered tomograms obtained by imaging comet tails in cytoskeletons over holes in perforated films, to reduce background noise. As we show, useful complementary structural information was also provided from electron tomography of negatively stained samples due to higher image contrast and filament detail. Taking into account these different factors we have been able to provide the first 3D structure, to our knowledge, of an actin comet tail propelling a pathogen. Our demonstration of a minimal actin filament cassette for propulsion provides a new framework for analyzing and modeling the underlying mechanisms of actin force production by Arp2/3-complex-dependent actin assemblies.

The actin-based propulsion of loads (from *L. monocytogenes* to beads) has already attracted the interest of several groups [16],. In the case of the baculovirus tail with its limited number of filaments, we can exclude the squeezing mechanism proposed in the active gel model [23]. Mogilner and Oster [25] and Dickinson and Purich [26] provide useful alternative models of how actin filaments may push at the pathogen surface disregarding actin tail architecture. Alberts and Odell's [27] comprehensive *in silico* model simulates details of the actin filament organization but without knowledge of the actual structure. We chose a tractable modeling approach (see Text S1) using a minimal number of parameters (Table S1) to define the essential set of mechanisms required to simulate the observed filament organization and the motion of the virus *in vivo*.

By comparing alternative mathematical simulations with our experimental data, we may propose key mechanistic features of comet tail propulsion. In particular, comet tail organization and the trajectories of the virus were best explained by assuming continuous attachment of actin filament to the virus surface, supporting the hypothesis that tethering, possibly by VASP (Figure 1B) [28], which is present in the baculovirus tail, and pulling forces between the tail and the pathogen [17],[29] are needed to stabilize viral movement. A critical parameter influencing the modeling outcome was Brownian motion (see Text S1). Without it, there was little difference between the simulations for tethered and un-tethered filaments. Since the virus must experience external jostling forces in the cytoplasm, we consider the model featuring Brownian motion and tethering to mimic more closely the situation *in vivo*. An interesting feature of the model was the prediction that nucleation of filaments on the virus is required not only to initiate movement, but also intermittently to correct sharp turns and a stochastic, regional paucity of pushing filaments. As a consequence, sequential subsets of branched filaments are created, consistent with the tomography data and reminiscent of the organization of actin filaments in lamellipodia [12]. The fishbone-like array of filaments in the comet tail could only be simulated if branching was biased toward the virus surface with the result that all new filaments are involved in pushing. The viral nucleation promoting factor, p78/83, therefore seems to restrict branching from tethered mother filaments in a biased way, through conformational constraints, or possibly as a result of local bending of actin filaments at the filament–virus interface [30]. The delay of capping protein incorporation in the comet tail corresponding to around 1 s (Figure 1B) is consistent with the observed filament length distribution ranging from 10 nm to 800 nm (Figures 2, 3, and 6K). The later incorporation of cofilin (Figure 1B) is likewise consistent with its preferential binding to ADP-F-actin [31].

Fishbone-like patterns of actin have been described in comet tails induced in cells by vaccinia virus [32] as well as by *L. monocytogenes* in *in vitro* motility assays [5] at low gelsolin concentrations. The principles of organization of actin filaments in the baculovirus actin comet tail shown here thus likely apply generally to comets formed on pathogens that hijack the Arp2/3 complex machinery for propulsion and invasion [6].

## Methods

### Virus Preparation

The supernatant from Sf9 cells infected with either wild-type or mCherry-tagged baculovirus [14] was harvested after 3–5 d, precleared at 2,500 g for 5 min, filtered through a membrane with 0.45 µm pore size, then centrifuged at 18,000 g for 1 h and re-suspended in 150 µl of baculovirus buffer (10 mM HEPES, 0.5 M KCl, pH 7.4) to avoid clustering of the viruses. For *in vitro* assays, de-enveloped viruses were prepared by adding 2% Triton X-100 (Fluka) to the virus suspension and incubating the mixture on a shaker for 15 min at 25°C. The suspension was then centrifuged at 500 rpm to remove large debris and the supernatant centrifuged at 20,000 g for 1 h. The resulting pellet was resuspended in 50 µl baculovirus buffer supplemented with 1% BSA and used as stock in the motility assay.

### *In Vitro* Motility Assay

The *in vitro* assay was basically performed as described [4]. Briefly, ADF (3.7 µM), Profilin (2.5 µM), Gelsolin (25–200 nM), Arp2/3 (75 nM), G-Actin (7.6 µM), VASP (100 nM), and the purified, de-enveloped baculovirus were mixed in X-Buffer (10 mM HEPES, 100 mM KCl, 1 mM MgCl_2_, 0.1 mM CaCl_2_, pH 7.8) supplemented with 1% BSA, 2 mM ATP, 4 mM MgCl_2_, and 6.7 mM DTT. The motility assay was incubated for different times as a drop on a grid in a wet chamber at room temperature.

### Cell Culture

B16 mouse melanoma cells were cultured in Dulbecco's Modified Eagle's Medium (DMEM) (Sigma-Aldrich) with 10% fetal calf serum (Thermo Fisher Scientific), 1% penicillin/streptomycin, and 1% L-glutamine. B16 cells were transfected as subconfluent monolayer cultures in 30 mm Petri dishes using 2 µg of DNA, 100 µl Optimem (Invitrogen), and 6 µl Fugene (Roche). Before addition to the cells overnight, the transfection mix was incubated for 20 min. The next day, the medium was changed and the transfected cells plated on coverslips coated with 25 µg/ml laminin (Sigma) for fluorescence microscopy. Sf9 cells were kept in Grace's insect medium with 10% fetal bovine serum, 1% penicillin/streptomycin, and 1% L-glutamine. Goldfish fin fibroblasts (line CAR, No. CCL71, ATCC) were maintained in Basal Eagle's Medium (Sigma-Aldrich) supplemented with 1% nonessential amino acids, 2.5% HEPES (Invitrogen), 1% penicillin/streptomycin, 1% L-glutamine, and 12% fetal bovine serum (Thermo Fisher Scientific) at 27°C. For transient transfection, subconfluent monolayer cultures on 30 mm Petri dishes were used. The transfection mixture was prepared as follows: 2 µg of DNA and 12 µl of Superfect lipofection agent (Qiagen) were mixed in 200 µl Optimem (Invitrogen). After 15 min incubation at room temperature, a further 5% serum-containing medium with transfection mixture was added to the cells for 5 h. The cells were then washed and returned to normal medium. Transfected cells were replated after 24 h on 15 mm coverslips coated with human fibronectin (Roche) at a concentration of 50 µg/ml.

For virus uptake 150 µl baculovirus stock was added to 2 ml of a subconfluent cell culture growing on coated coverslips or electron microscopy grids and incubated at 27°C or 37°C depending on the cell type, and imaging was performed after 1–3 h.

Plasmids used for transfection were mCherry-actin [33], pEGFP-actin (Clontech), pEGFP-ArpC5 [34], pEGFP-VASP [35], pEGFP-Capping Protein β2 [36], and mCherry-Cofilin [37]. Baculovirus particles tagged with mCherry were prepared as described [14].

### Live Cell Imaging and Analysis

Coverslips carrying baculovirus-infected cells were mounted in an open chamber on a heating platform (Harvard Instruments) in prewarmed medium. Intracellular virus-induced tails were usually observed after about 20 min postinfection. The samples were observed on an inverted Zeiss Observer epifluorescence microscope equipped with a Perkin Elmer UltraView spinning disc system (ProSync2). For kymograph analysis, Fiji together with a MatLab-based script were used. Statistical analysis was performed using Prism. Intensity values of tail components were normalized to tail length based on the linear relationship between tail length and speed (Figure S1E).

### Electron Microscopy

For negative staining electron microscopy, cells were grown on Formvar-coated 200 mesh hexagonal nickel grids (Agar Scientific) or 135 mesh NHF15-A gold Finder grids (Maxtaform) in standard medium and allowed to spread overnight. After spreading, the samples were washed with cytoskeleton buffer (10 mM MES buffer, 150 mM NaCl, 5 mM EGTA, 5 mM glucose, 5 mM MgCl_2_, pH 6.8), fixed and extracted with 0.5% Triton X-100 and 0.25% glutaraldehyde in cytoskeleton buffer for 1 min, and kept in 2% glutaraldehyde with 1 µg/ml phalloidin in cytoskeleton buffer at 4°C. For routine inspection in an 80 kV transmission electron microscope (Morgagni, FEI), grids were stained with 70 µl 2% SST including 1 µg/ml phalloidin. For electron tomography, grids were stained with 70 µl 6%–8% sodium silicotungstate (SST) including 1 µg/ml phalloidin. For cryo-electron tomography, virus-infected cells were plated in growth medium onto Quantifoil R1/4, R1.2/1.3, and R2/2 perforated carbon films on 200 mesh Au grids, and allowed to spread for 6 h. The cells were fixed as for negative staining and the grids subsequently transferred to forceps in a grid-plunging device (Leica EM GP), supplemented with 4 µl of medium and 10 nm BSA-saturated colloidal gold [12]. During mounting and blotting, the grid was held in a chamber with controlled humidity and temperature (95% and 22°C) and was blotted automatically for 1.5–2 s with humidified Whatman No. 1 filter paper applied to the backside of the grid to avoid any contact with the cells. Samples were frozen by plunging into liquid ethane cooled to 80 K by liquid nitrogen. *In vitro* polymerized actin tails were prepared for electron microcopy as follows. For negative staining the incubation mix was fixed after different times on the 200 mesh Au grids by injecting 2% glutaraldehyde (final concentration 0.5%) for 4 min and stained with 70 µl 2% sodium silicotungstate (SST). For cryo-electron microscopy, samples were incubated on Quantifoil R3.5/1 perforated carbon films on 200 mesh Au grids for 45 min, rinsed with 70 µl X-Buffer, followed by 70 µl X-Buffer with 1∶10 BSA-colloid gold, and without fixation or fixed as above, frozen as described above with blotting times between 1.2 and 1.6 s. Tilt series were acquired on an FEI Tecnai F30 Helium (Polara) microscope, operated at 300 kV, and cooled to approximately 80 K. Automated acquisition of tilt series was driven by SerialEM 3.x. Normally, the tilt range was −60° to +60° using the Saxton tilt scheme based on 1° increments for negative stain and 2° increments for cryo-samples from 0° tilt, at a nominal defocus value of −5 µm to −7 µm for negative stain and −8 µm to −12 µm for cryo-samples. For negative stain samples, two tilt series around orthogonal axes were recorded on a Gatan UltraScan 4000 CCD camera at on-camera magnifications typically from 23,000× to 59,000×. For frozen hydrated samples, zero-loss images were recorded on a Gatan MSC 2 k camera on a GIF 2002 using a slit width of 15 eV. The total electron dose for the cryo-tilt series was less than 130 electrons per Å^2^, and the primary on-screen magnification was from 20,000× to 29,000×.

### Image Processing and Analysis

Re-projections from the tilt series with gold particles as fiducials for alignment and contrast transfer function corrections were performed using IMOD software [38]. A typical tomogram comprised a z-stack of 60–120 sections of 0.4–1.5 nm each. The polarity of actin filaments in tomograms of negatively stained and frozen samples was determined using a filament straightening protocol and cross-correlation analysis as described elsewhere [20]. Filaments were manually tracked using IMOD, as previously described [12].

The identity of branch junctions was based on filament tracking analysis, performed by three investigators independently. Filaments were tracked individually and branch junctions identified as blunt intersections of filament pairs subtending an acute angle in the range of 60–90°, as deduced for branch junctions in lamellipodia [12]. An example of the tracking analysis in the cryo-tomograms is shown in Movie S4.

### Mathematical Modeling

Mathematical modeling was performed using MatLab software. Details of the model are provided in Text S1.

## Supporting Information

Figure S1 Determination of relative distribution of components along baculovirus comet tails. (A) Series of time-lapse images taken at 3 s intervals. (B) Kymograph along the tail trajectory. Red arrows indicate direction of intensity measurements. (C) Average of 20 measurements along the tail shown in (A). To average signals along the comet tail trajectories, the intensity value of the background around 1 µm in front of the virus was subtracted from all measurements along the trajectory. The resulting data were normalized to maximal intensity, plotted on the *y* axis, and to tail length, plotted on the *x* axis. At the position of the virus, defined by the mCherry-label (*n* = 689 measurements in 26 individual tails), the GFP-actin intensity corresponded to 0.15 times the maximal intensity. This intensity value was used to define the beginning and end of the tail, in the case of actin (*n* = 2,776 in 111 tails). For VASP (*n* = 929 in 30 tails), ArpC5 (*n* = 300 in 20 tails), capping protein β (*n* = 313 in 13 tails), and cofilin (*n* = 395 in 17 tails) co-labeling with fluorescent actin was used in the same way to determine the limits of the comet tail. (D) Images of comet tails showing the localization of the proteins indicated (red arrowheads indicate front). (E) Relationship of tail length to virus speed (*n* = 111). Correlation **** (Pearson constant = 0.8652, *p*<0.0001). Bars, (A) 3 µm, (B, D) 1 µm (horizontally) and 20 s (vertically).
(TIF)

Figure S2 Actin comet tails are preserved after fixation in a mixture of glutaraldehyde and Triton X-100. (A) Video frames of a CAR fibroblast that was infected with mCherry-tagged baculovirus and transfected with GFP-actin (0–48 s). Thereafter the cell was immediately extracted with a glutaraldehyde/Triton mixture (Extracted). Red arrowheads indicate position of the virus at the head of the comet tails, marked 1 and 2 in the “Extracted” frame. (B) Plots of the normalized GFP fluorescence intensity along the two comet tails indicated just before (48 s) and after extraction. Plots in red indicate fluorescence intensity in the mCherry channel. Bar, 2 µm.
(TIF)

Figure S3 Determination of actin filament polarity in the comet tail shown in Figure 2. (A) Isosurface rendering of comet tail showing filaments analyzed in red. (B) Analyzed filaments highlighted against the core of the comet tail. (C) Straightened filaments from numbered positions in (A) and (B), with the plus and minus ends marked according to the analysis in (D). (D) Cross-correlation analysis of actin polarity of the marked filaments. Twenty-four of the 27 filaments pointed toward the baculovirus. Filaments 2, 21, and 26 marked as oriented in the opposite direction are located in regions of tightly packed actin, where polarity determination is less certain. y_i_ indicates position along the filament axis. Bars, 100 nm.
(TIF)

Figure S4 Examples of additional tomograms of comet tails *in vivo*. Examples of actin comet tails in cytoskeletons embedded in negative stain together with the filament trajectories derived from the tomograms. The images correspond to a combination of 10–15 Z-stacks in the corresponding tomogram series. Bars, 100 nm.
(TIF)

Figure S5 Further examples of negatively stained and cryo tomograms of comet tails *in vivo*. Figure shows actin comet tails in cytoskeletons embedded in negative stain (A–C) and in vitreous ice (D and E) together with the filament trajectories derived from the tomograms. The images correspond to a combination of 10–20 Z-stacks in the corresponding tomogram series. Bars, 100 nm.
(TIF)

Figure S6 Different views of a cryo-tomogram model of a comet tails *in vivo*. Top and end-on views of the tracked filament trajectories in a tomogram of a baculovirus comet tail in vitreous ice (corresponding to the tomogram shown in Figure 6D). The projections serve to illustrate the spatial segregation between the comet tail filaments (green) and the filaments of the host cell (grey). Bar, 200 nm.
(TIF)

Figure S7 Determination of actin filament polarity in a cryo-electron tomogram of a comet tail formed *in vitro*. (A) Section of tomogram (19 nm). (B) Model showing numbered filaments analyzed with barbed ends marked by black spheres. (C) Straightened filaments with the plus and minus ends obtained by cross-correlation analysis with the reference filaments. (D) Typical filament before and after image processing by signal enhancement around 5.0 nm and low-pass filtering at 3.7 nm. (E) Cross-correlation plots of filaments analyzed. y_i_ indicates position along the filament axis. (F) Sequence of analysis steps. Bars, 100 nm.
(TIF)

Movie S1 Live cell imaging of baculovirus actin comets. (Part 1) Actin comet tails in mCherry-baculovirus-infected B16 melanoma cells transfected with GFP actin. (Part 2) Examples of baculovirus-induced actin comet tails in B16 cells transfected with the indicated proteins, as used for kymograph analysis (see Figures 1B and S1). Heads of comet tails are marked by red arrows.
(MOV)

Movie S2 Electron tomogram of negatively stained comet tail *in vivo*. Scan of electron tomogram of negatively stained comet tail from Figure 2A and the model derived by manual tracking in Figure 2B. Actin filament subsets are highlighted at the end of the movie.
(MOV)

Movie S3 Cryo-electron tomogram of comet tail *in vivo*. Scan of electron tomogram of comet tail in vitreous ice from Figure 3A and the model derived by manual tracking in Figure 3B,D. Actin filaments, first in green, are highlighted as subsets in different colors in the middle of the movie. Branch points are in red. Actin filaments belonging to the cytoskeleton of the cell are indicated in grey, as is also a microtubule crossing the field. At the end of the movie, the pushing core of the tail is highlighted in yellow.
(MOV)

Movie S4 Example of the filament tracking procedure in cryo-electron tomograms. Movie shows Z-scan through same tomogram as Movie S3, but with the manual tracking of several filament segments in the second part. Red spheres indicate the branch junctions and blue spheres individual tracking points.
(MOV)

Movie S5 Cryo-electron tomogram of comet tail assembled *in vitro*. Scan of electron tomogram of *in vitro* comet tail in vitreous ice from Figure 5A and the model derived by manual tracking in Figure 5B,C. Actin filaments are in green and branch junctions in red. The end of the movie shows the actin subset abutting the virus, with four filaments associated with the virus rear.
(MOV)

Movie S6 Simulation of baculovirus movement with and without filament tethering. (Part 1) Simulation with filaments tethered to the virus. The beginning of Part 1 shows initiation of filament subsets, in different colors, linked by branch junctions in red, followed by debranching (transparent points) and depolymerization of actin filaments at the rear. Subsequently, some filament subsets and branches are highlighted, followed by an overview of the tracking course marked by a dashed line. (Part 2) Same input parameters as in Part 1 except with untethered filaments. Subsets are marked in different colors.
(MOV)

Table S1 Model input parameters.
(DOCX)

Table S2 Matched parameters of the simulation assuming continuous tethering of filaments to the virus surface compared to experimentally observed parameters.
(DOCX)

Table S3 Predicted parameters of the simulation assuming continuous tethering of filaments to the virus surface compared to experimentally observed parameters.
(DOCX)

Text S1 Details of mathematical simulation.
(DOCX)

## Abbreviations

ActA: Actin assembly-inducing protein
ADF: Actin-depolymerizing factor
ADP: Adenosine diphosphate
Arp2/3 complex: Actin-related protein 2/3 complex
ArpC5: Actin-related protein C5
ATP: Adenosine triphosphate
BSA: Bovine serum albumin
CCD: charge-coupled device
DTT: dithiothreitol
GFP: green fluorescent protein
HEPES: 4-(2-hydroxyethyl)-1-piperazineethanesulfonic acid
N-WASP: Neuronal Wiskott–Aldrich syndrome protein
VASP: Vasodilator-stimulated phosphoprotein

## References

[1] Tilney LG, Portnoy DA (1989) Actin filaments and the growth, movement, and spread of the intracellular bacterial parasite, Listeria monocytogenes. J Cell Biol 109: 1597–1608. 2507553 10.1083/jcb.109.4.1597 PMC2115783

[2] Machesky LM, Atkinson SJ, Ampe C, Vandekerckhove J, Pollard TD (1994) Purification of a cortical complex containing two unconventional actins from Acanthamoeba by affinity chromatography on profilin-agarose. J Cell Biol 127: 107–115. 7929556 10.1083/jcb.127.1.107 PMC2120189

[3] Welch MD, Iwamatsu A, Mitchison TJ (1997) Actin polymerization is induced by Arp2/3 protein complex at the surface of Listeria monocytogenes. Nature 385: 265–269. 9000076 10.1038/385265a0

[4] Loisel TP, Boujemaa R, Pantaloni D, Carlier MF (1999) Reconstitution of actin-based motility of Listeria and Shigella using pure proteins. Nature 401: 613–616. 10524632 10.1038/44183

[5] Wiesner S, Helfer E, Didry D, Ducouret G, Lafuma F, et al (2003) A biomimetic motility assay provides insight into the mechanism of actin-based motility. J Cell Biol 160: 387–398. 12551957 10.1083/jcb.200207148 PMC2172664

[6] Goley ED, Welch MD (2006) The ARP2/3 complex: an actin nucleator comes of age. Nat Rev Mol Cell Biol 7: 713–726. 16990851 10.1038/nrm2026

[7] Dodding MP, Way M (2009) Nck- and N-WASP-dependent actin-based motility is conserved in divergent vertebrate poxviruses. Cell Host Microbe 6: 536–550. 20006842 10.1016/j.chom.2009.10.011

[8] Haglund CM, Welch MD (2011) Pathogens and polymers: microbe–host interactions illuminate the cytoskeleton. J Cell Biol 195: 7–17. 21969466 10.1083/jcb.201103148 PMC3187711

[9] Sechi AS, Wehland J, Small JV (1997) The isolated comet tail pseudopodium of Listeria monocytogenes: a tail of two actin filament populations, long and axial and short and random. J Cell Biol 137: 155–167. 9105044 10.1083/jcb.137.1.155 PMC2139863

[10] Cameron LA, Svitkina TM, Vignjevic D, Theriot JA, Borisy GG (2001) Dendritic organization of actin comet tails. Curr Biol 11: 130–135. 11231131 10.1016/s0960-9822(01)00022-7

[11] Gouin E, Welch MD, Cossart P (2005) Actin-based motility of intracellular pathogens. Curr Opin Microbiol 8: 35–45. 15694855 10.1016/j.mib.2004.12.013

[12] Vinzenz M, Nemethova M, Schur F, Mueller J, Narita A, et al (2012) Actin branching in the initiation and maintenance of lamellipodia. J Cell Sci 125: 2775–2785. 22431015 10.1242/jcs.107623

[13] Goley ED, Ohkawa T, Mancuso J, Woodruff JB, D'Alessio JA, et al (2006) Dynamic nuclear actin assembly by Arp2/3 complex and a baculovirus WASP-like protein. Science 314: 464–467. 17053146 10.1126/science.1133348

[14] Ohkawa T, Volkman LE, Welch MD (2010) Actin-based motility drives baculovirus transit to the nucleus and cell surface. J Cell Biol 190: 187–195. 20660627 10.1083/jcb.201001162 PMC2930276

[15] Machesky LM, Insall RH, Volkman LE (2001) WASP homology sequences in baculoviruses. Trends Cell Biol 11: 286–287. 11434350 10.1016/s0962-8924(01)02009-8

[16] Mogilner A (2009) Mathematics of cell motility: have we got its number? J Math Biol 58: 105–134. 18461331 10.1007/s00285-008-0182-2 PMC2862828

[17] Dickinson RB (2009) Models for actin polymerization motors. J Math Biol 58: 81–103. 18612640 10.1007/s00285-008-0200-4

[18] Urban E, Jacob S, Nemethova M, Resch GP, Small JV (2010) Electron tomography reveals unbranched networks of actin filaments in lamellipodia. Nat Cell Biol 12: 429–435. 20418872 10.1038/ncb2044

[19] Narita A, Maeda Y (2007) Molecular determination by electron microscopy of the actin filament end structure. J Mol Biol 365: 480–501. 17059832 10.1016/j.jmb.2006.06.056

[20] Narita A, Mueller J, Urban E, Vinzenz M, Small JV, et al (2012) Direct determination of actin polarity in the cell. J Mol Biol 419: 359–368. 22459261 10.1016/j.jmb.2012.03.015 PMC3370650

[21] Lacayo CI, Soneral PA, Zhu J, Tsuchida MA, Footer MJ, et al (2012) Choosing orientation: influence of cargo geometry and ActA polarization on actin comet tails. Mol Biol Cell 23: 614–629. 22219381 10.1091/mbc.E11-06-0584 PMC3279390

[22] Dickinson RB (2008) A multi-scale mechanistic model for actin-propelled bacteria. Cellular and Molecular Bioengineering 1: 110–121.

[23] Plastino J, Sykes C (2005) The actin slingshot. Curr Opin Cell Biol 17: 62–66. 15661520 10.1016/j.ceb.2004.12.001

[24] Upadhyaya A, Chabot JR, Andreeva A, Samadani A, van Oudenaarden A (2003) Probing polymerization forces by using actin-propelled lipid vesicles. Proc Natl Acad Sci U S A 100: 4521–4526. 12657740 10.1073/pnas.0837027100 PMC153588

[25] Mogilner A, Oster G (2003) Force generation by actin polymerization II: the elastic ratchet and tethered filaments. Biophys J 84: 1591–1605. 12609863 10.1016/S0006-3495(03)74969-8 PMC1302730

[26] Dickinson RB, Purich DL (2002) Clamped-filament elongation model for actin-based motors. Biophys J 82: 605–617. 11806905 10.1016/S0006-3495(02)75425-8 PMC1301872

[27] Alberts JB, Odell GM (2004) In silico reconstitution of Listeria propulsion exhibits nano-saltation. PLoS Biol 2: e412. 15562315 10.1371/journal.pbio.0020412 PMC532387

[28] Breitsprecher D, Kiesewetter AK, Linkner J, Vinzenz M, Stradal TE, et al (2011) Molecular mechanism of Ena/VASP-mediated actin-filament elongation. EMBO J 30: 456–467. 21217643 10.1038/emboj.2010.348 PMC3034019

[29] Tsuchida MA, Theriot JA (2007) Decoupling the coupling: surface attachment in actin-based motility. ACS Chem Biol 2: 221–224. 17455897 10.1021/cb700071d

[30] Risca VI, Wang EB, Chaudhuri O, Chia JJ, Geissler PL, et al (2012) Actin filament curvature biases branching direction. Proc Natl Acad Sci U S A 109: 2913–2918. 22308368 10.1073/pnas.1114292109 PMC3286980

[31] Carlier MF, Laurent V, Santolini J, Melki R, Didry D, et al (1997) Actin depolymerizing factor (ADF/cofilin) enhances the rate of filament turnover: implication in actin-based motility. J Cell Biol 136: 1307–1322. 9087445 10.1083/jcb.136.6.1307 PMC2132522

[32] Cudmore S, Reckmann I, Griffiths G, Way M (1996) Vaccinia virus: a model system for actin-membrane interactions. J Cell Sci 109 Pt 7: 1739–1747. 8832396 10.1242/jcs.109.7.1739

[33] Nemethova M, Auinger S, Small JV (2008) Building the actin cytoskeleton: filopodia contribute to the construction of contractile bundles in the lamella. J Cell Biol 180: 1233–1244. 18362182 10.1083/jcb.200709134 PMC2290848

[34] Lai FP, Szczodrak M, Block J, Faix J, Breitsprecher D, et al (2008) Arp2/3 complex interactions and actin network turnover in lamellipodia. EMBO J 27: 982–992. 18309290 10.1038/emboj.2008.34 PMC2265112

[35] Rottner K, Behrendt B, Small JV, Wehland J (1999) VASP dynamics during lamellipodia protrusion. Nat Cell Biol 1: 321–322. 10559946 10.1038/13040

[36] Schafer DA, Welch MD, Machesky LM, Bridgman PC, Meyer SM, et al (1998) Visualization and molecular analysis of actin assembly in living cells. J Cell Biol 143: 1919–1930. 9864364 10.1083/jcb.143.7.1919 PMC2175235

[37] Breitsprecher D, Koestler SA, Chizhov I, Nemethova M, Mueller J, et al (2011) Cofilin cooperates with fascin to disassemble filopodial actin filaments. J Cell Sci 124: 3305–3318. 21940796 10.1242/jcs.086934 PMC4074248

[38] Kremer JR, Mastronarde DN, McIntosh JR (1996) Computer visualization of three-dimensional image data using IMOD. J Struct Biol 116: 71–76. 8742726 10.1006/jsbi.1996.0013

