# Supplementary figures and images for: Electron Tomography and Simulation of Baculovirus Actin Comet Tails Support a Tethered Filament Model of Pathogen Propulsion
Source: PLoS Biol. 2014 Jan 14;12(1):e1001765. doi: 10.1371/journal.pbio.1001765 (PMC3891563; doi:10.1371/journal.pbio.1001765)

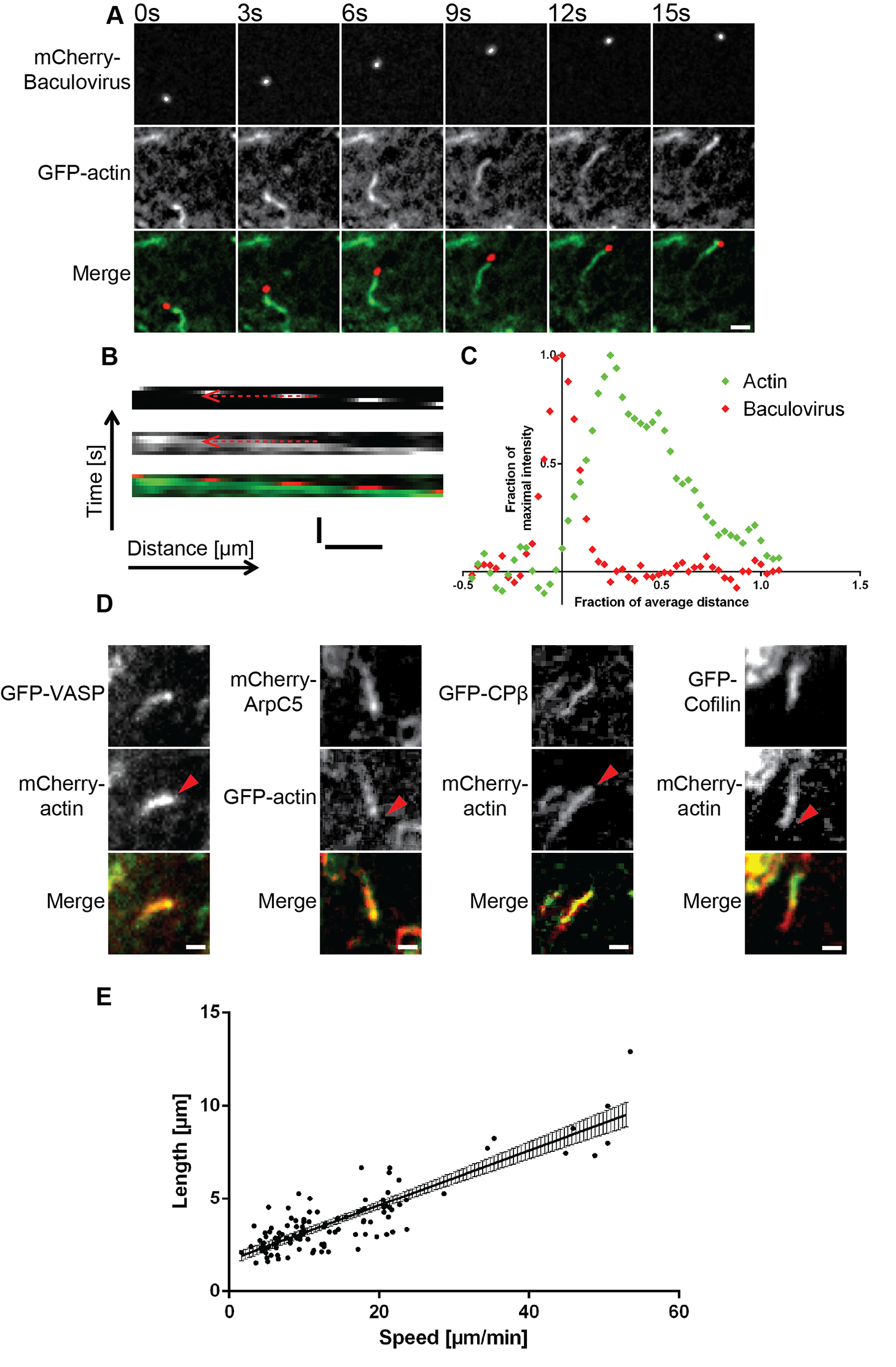

Supplement: Figure S1 — Determination of relative distribution of components along baculovirus comet tails. (A) Series of time-lapse images taken at 3 s intervals. (B) Kymograph along the tail trajectory. Red arrows indicate direction of intensity measurements. (C) Average of 20 measurements along the tail shown in (A). To average signals along the comet tail trajectories, the intensity value of the background around 1 µm in front of the virus was subtracted from all measurements along the trajectory. The resulting data were normalized to maximal intensity, plotted on the y axis, and to tail length, plotted on the x axis. At the position of the virus, defined by the mCherry-label (n = 689 measurements in 26 individual tails), the GFP-actin intensity corresponded to 0.15 times the maximal intensity. This intensity value was used to define the beginning and end of the tail, in the case of actin (n = 2,776 in 111 tails). For VASP (n = 929 in 30 tails), ArpC5 (n = 300 in 20 tails), capping protein β (n = 313 in 13 tails), and cofilin (n = 395 in 17 tails) co-labeling with fluorescent actin was used in the same way to determine the limits of the comet tail. (D) Images of comet tails showing the localization of the proteins indicated (red arrowheads indicate front). (E) Relationship of tail length to virus speed (n = 111). Correlation **** (Pearson constant = 0.8652, p<0.0001). Bars, (A) 3 µm, (B, D) 1 µm (horizontally) and 20 s (vertically). (TIF) [file pbio.1001765.s001.tif]

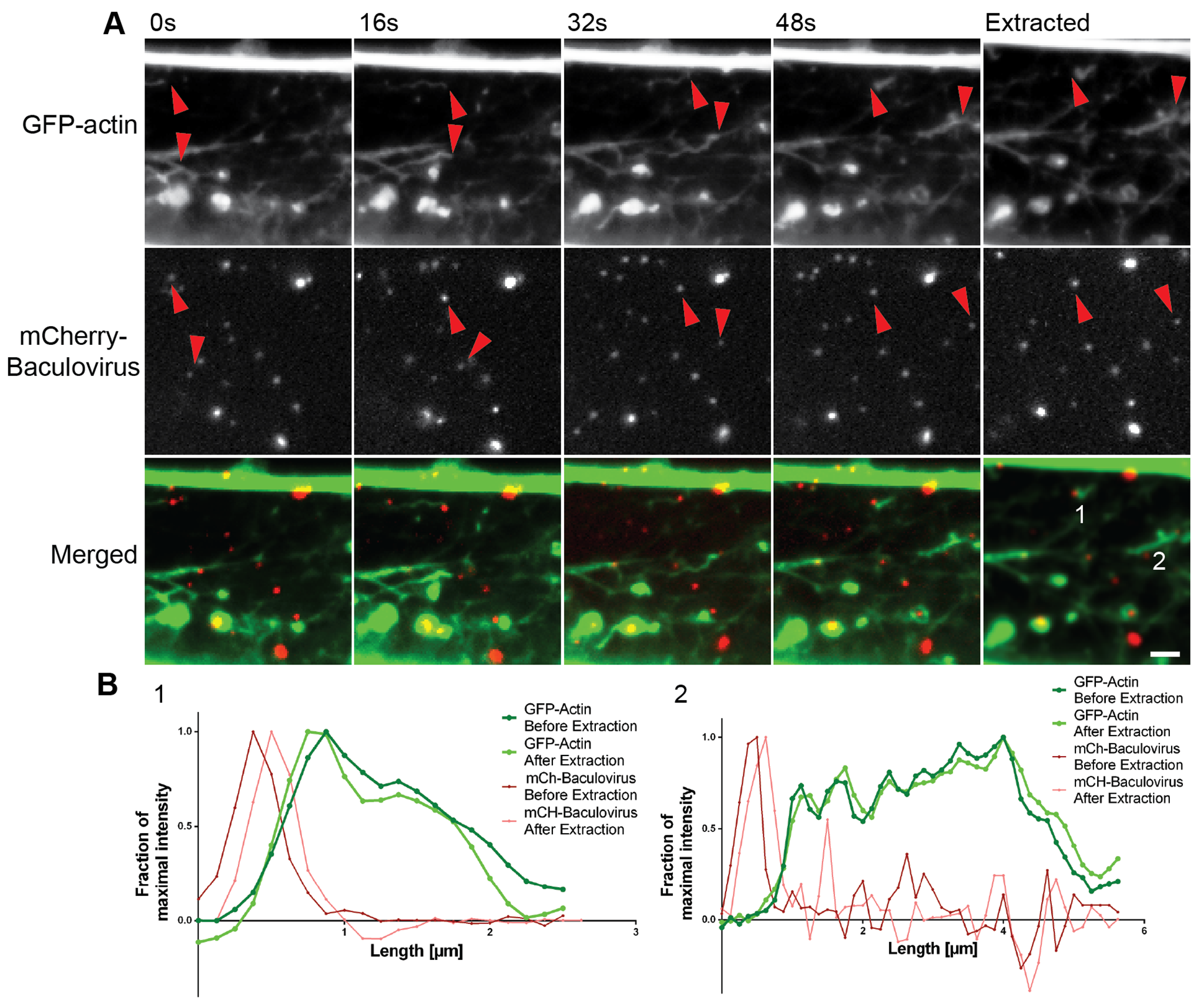

Supplement: Figure S2 — Actin comet tails are preserved after fixation in a mixture of glutaraldehyde and Triton X-100. (A) Video frames of a CAR fibroblast that was infected with mCherry-tagged baculovirus and transfected with GFP-actin (0–48 s). Thereafter the cell was immediately extracted with a glutaraldehyde/Triton mixture (Extracted). Red arrowheads indicate position of the virus at the head of the comet tails, marked 1 and 2 in the “Extracted” frame. (B) Plots of the normalized GFP fluorescence intensity along the two comet tails indicated just before (48 s) and after extraction. Plots in red indicate fluorescence intensity in the mCherry channel. Bar, 2 µm. (TIF) [file pbio.1001765.s002.tif]

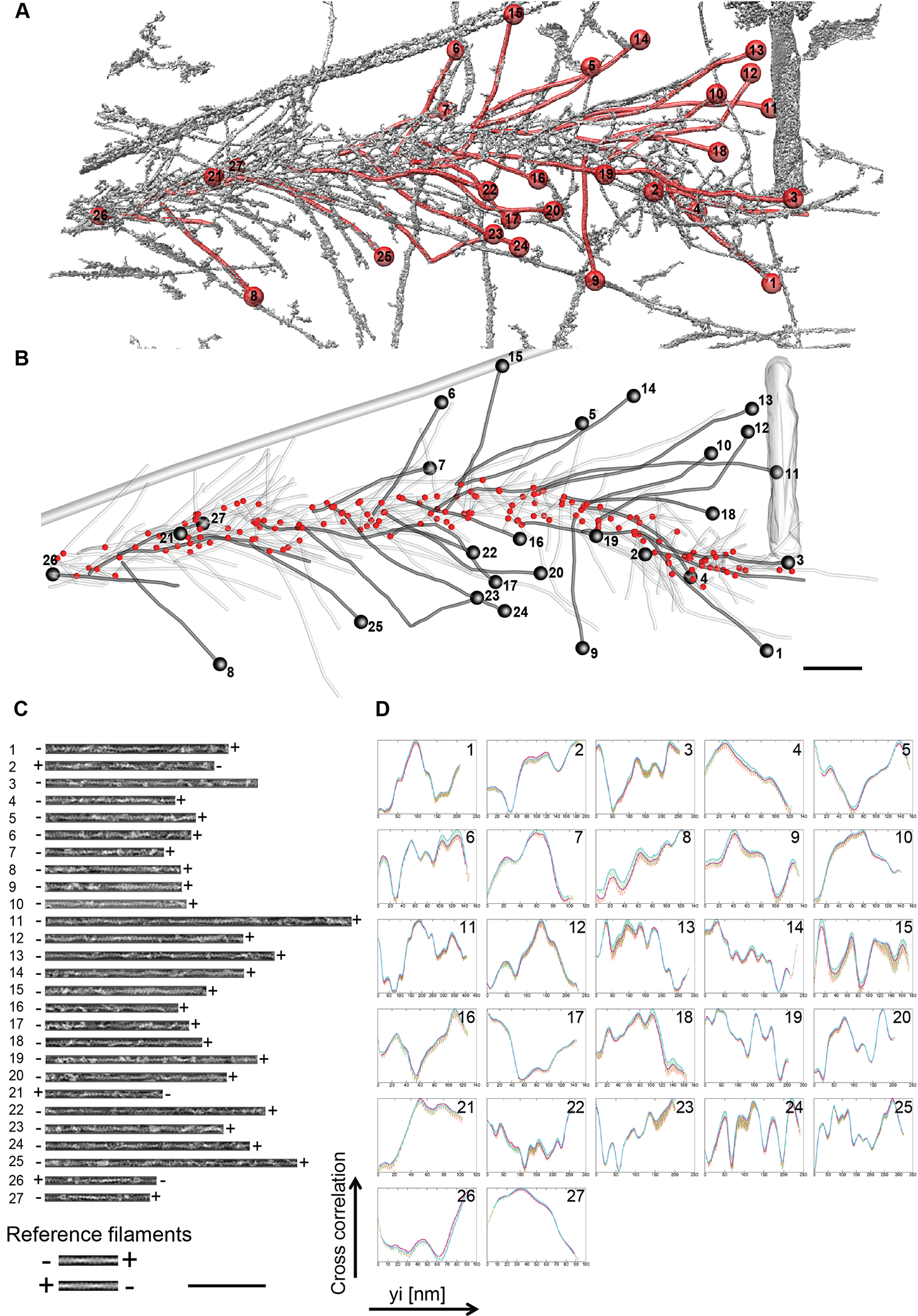

Supplement: Figure S3 — Determination of actin filament polarity in the comet tail shown in Figure 2. (A) Isosurface rendering of comet tail showing filaments analyzed in red. (B) Analyzed filaments highlighted against the core of the comet tail. (C) Straightened filaments from numbered positions in (A) and (B), with the plus and minus ends marked according to the analysis in (D). (D) Cross-correlation analysis of actin polarity of the marked filaments. Twenty-four of the 27 filaments pointed toward the baculovirus. Filaments 2, 21, and 26 marked as oriented in the opposite direction are located in regions of tightly packed actin, where polarity determination is less certain. yi indicates position along the filament axis. Bars, 100 nm. (TIF) [file pbio.1001765.s003.tif]

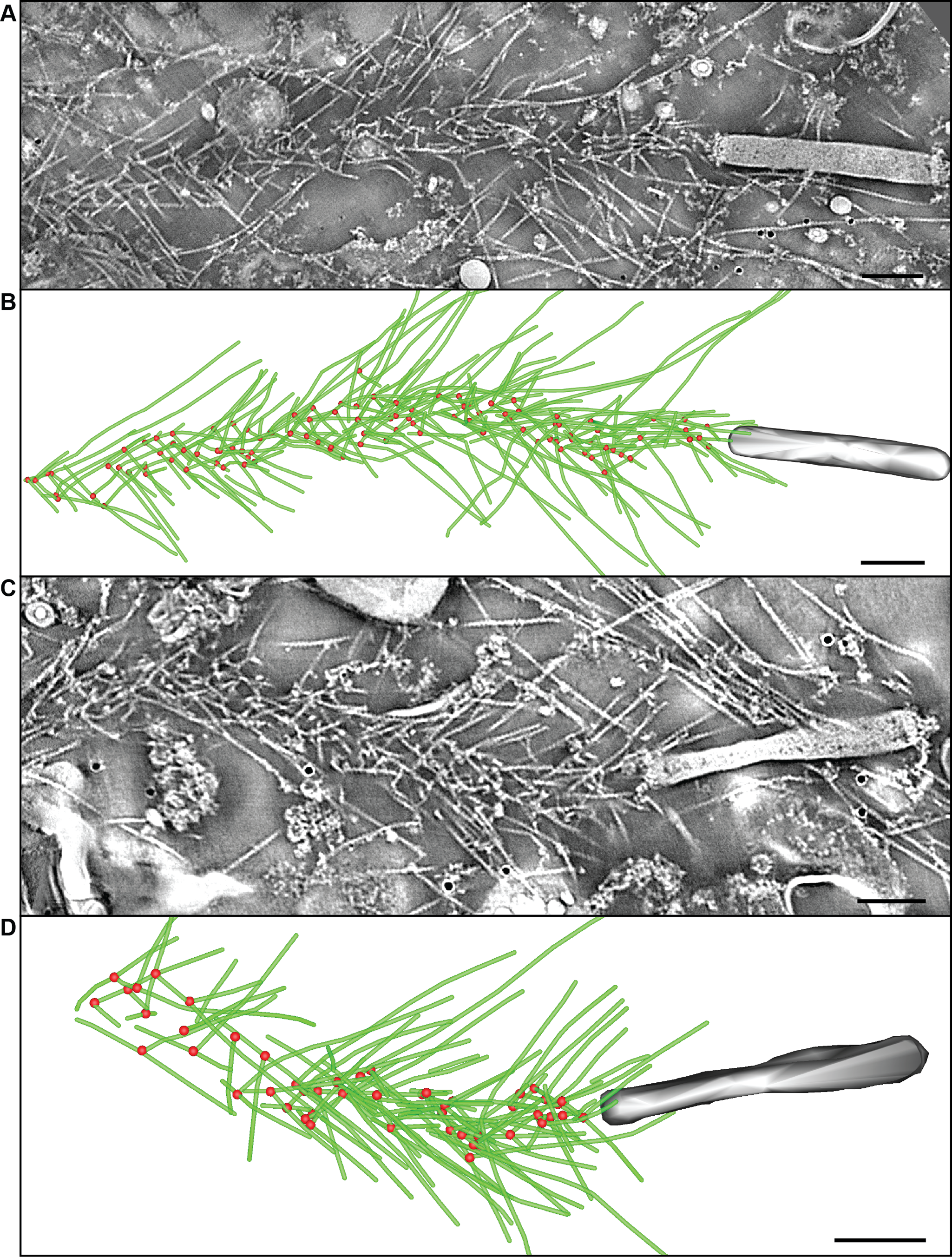

Supplement: Figure S4 — Examples of additional tomograms of comet tails in vivo. Examples of actin comet tails in cytoskeletons embedded in negative stain together with the filament trajectories derived from the tomograms. The images correspond to a combination of 10–15 Z-stacks in the corresponding tomogram series. Bars, 100 nm. (TIF) [file pbio.1001765.s004.tif]

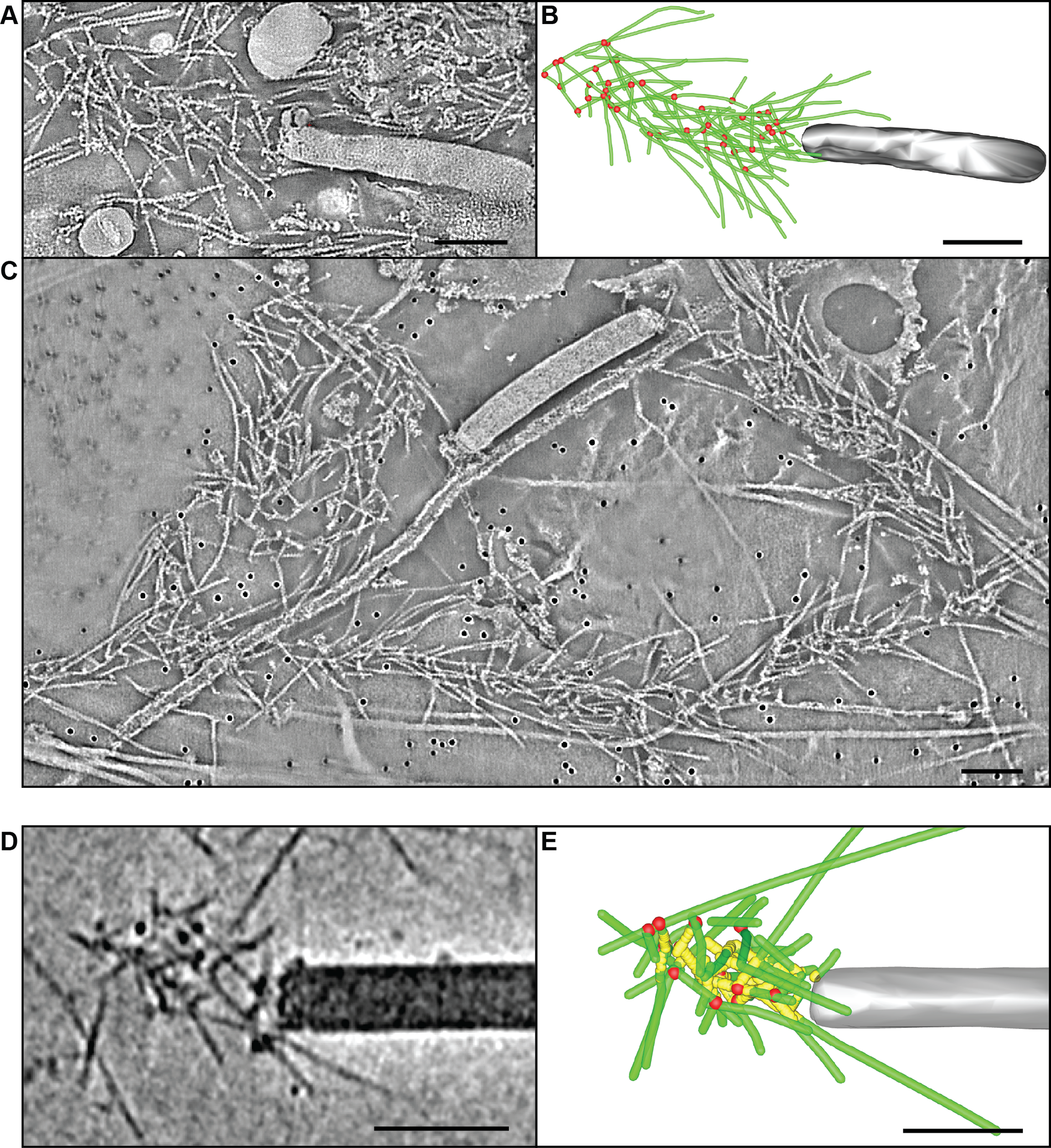

Supplement: Figure S5 — Further examples of negatively stained and cryo tomograms of comet tails in vivo. Figure shows actin comet tails in cytoskeletons embedded in negative stain (A–C) and in vitreous ice (D and E) together with the filament trajectories derived from the tomograms. The images correspond to a combination of 10–20 Z-stacks in the corresponding tomogram series. Bars, 100 nm. (TIF) [file pbio.1001765.s005.tif]

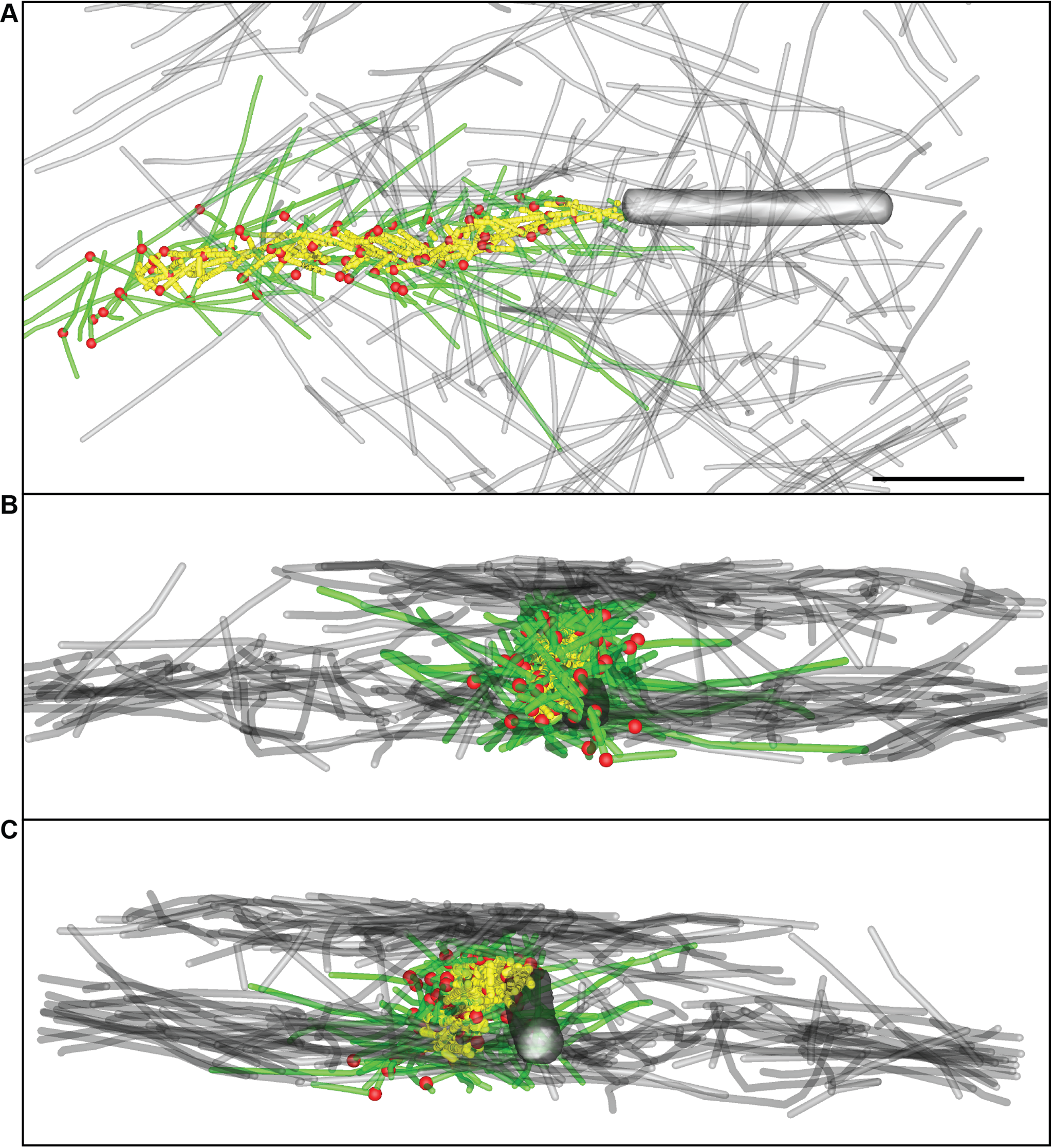

Supplement: Figure S6 — Different views of a cryo-tomogram model of a comet tails in vivo. Top and end-on views of the tracked filament trajectories in a tomogram of a baculovirus comet tail in vitreous ice (corresponding to the tomogram shown in Figure 6D). The projections serve to illustrate the spatial segregation between the comet tail filaments (green) and the filaments of the host cell (grey). Bar, 200 nm. (TIF) [file pbio.1001765.s006.tif]

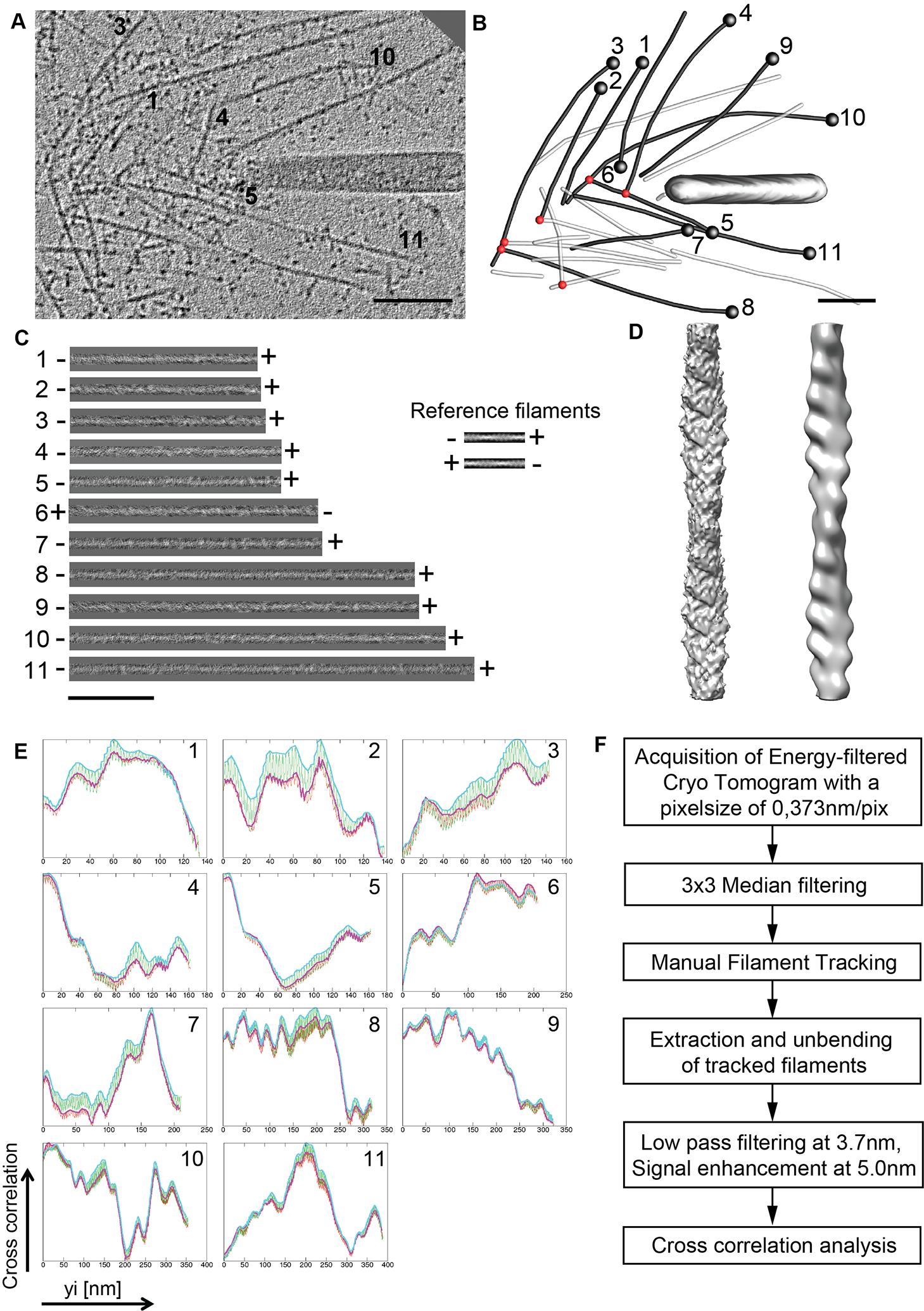

Supplement: Figure S7 — Determination of actin filament polarity in a cryo-electron tomogram of a comet tail formed in vitro. (A) Section of tomogram (19 nm). (B) Model showing numbered filaments analyzed with barbed ends marked by black spheres. (C) Straightened filaments with the plus and minus ends obtained by cross-correlation analysis with the reference filaments. (D) Typical filament before and after image processing by signal enhancement around 5.0 nm and low-pass filtering at 3.7 nm. (E) Cross-correlation plots of filaments analyzed. yi indicates position along the filament axis. (F) Sequence of analysis steps. Bars, 100 nm. (TIF) [file pbio.1001765.s007.tif]
