# Supplementary material for: Electron Tomography and Simulation of Baculovirus Actin Comet Tails Support a Tethered Filament Model of Pathogen Propulsion
Source: PLoS Biol. 2014 Jan 14;12(1):e1001765. doi: 10.1371/journal.pbio.1001765 (PMC3891563; doi:10.1371/journal.pbio.1001765)
Supplement: Table S1 — Model input parameters. (DOCX) [file pbio.1001765.s014.docx]

**Table S1:** Model Input parameters

| **Symbol** | **Meaning** | **Value** | **Justification** |
| --- | --- | --- | --- |
| $\beta_{max}$ | Describes the region on the virus surface, where p78/83 is present | 73.8° | Observed in EM [Supplementary reference 4] |
| $v_{max}$ | Maximal polymerization speed | 388nm*s^-1^ | Fitting parameter |
| $v_{min}$ | Minimal polymerization speed | 166nm*s^-1^ | Fitting parameter |
| $\gamma_{b}$ | Branching angle | 74° | Observed in ET |
| $\lambda_{b}$ | Maximal branching rate | 28nm*s^-1^ | Fitting parameter |
| $N$ | Maximal number of tethered filaments | 10 | Observed in ET |
| $\Delta\beta$ | Inhibition range of tethered barbed ends | 18° | Related to the filament diameter |
| $\lambda_{c}$ | Capping rate after detachment | 6.4s^-1^ | Fitting parameter |
| $\lambda_{d}$ | Rate of activation of depolymerization after capping and aging | 555s^-1^ | Fitting parameter (tail length) |
| $v_{d}$ | Depolymerization speed | 388nm | Fitting parameter (tail length) |
| $n_{0}$ | Threshold number of tethered barbed ends for activating nucleation | 4 | Observed in ET |
| $\lambda_{n}$ | Nucleation rate (if 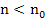n < n_0_) | 9.4s^-1^ | Fitting parameter |
| $L$ | Length of the rectangular part of the virus | 300nm | Observed in ET |
| $r$ | Radius of the semicircles forming the ends of the virus | 25nm | Observed in ET |
| $\mu_{friction}$ | Friction coefficient | 5*10^-6^g*s^-1^*nm^-2^ | Fitting parameter |
| $\mu_{steric}$ | Penalization of steric interactions | 1g*s^-2^*nm^-1^ | Compromise: accuracy vs. computability |
| $\mu_{tether}$ | Elastic constant of tethers | 0.7g*s^-2^ | Fitting parameter |
| $z_{tether}$ | Maximal stretching of tethers | 2.75nm | Fitting parameter |
| $\Delta x$ | Maximal translation per time step by Brownian forcing | 0.2nm | Small compared to published data, qualitative effect |
| $\Delta\alpha$ | Maximal rotation per time step by Brownian forcing | 1.62° | Small compared to published data, qualitative effect |
