# Supplementary material for: Electron Tomography and Simulation of Baculovirus Actin Comet Tails Support a Tethered Filament Model of Pathogen Propulsion
Source: PLoS Biol. 2014 Jan 14;12(1):e1001765. doi: 10.1371/journal.pbio.1001765 (PMC3891563; doi:10.1371/journal.pbio.1001765)
Supplement: Table S2 — Matched parameters of the simulation assuming continuous tethering of filaments to the virus surface compared to experimentally observed parameters. (DOCX) [file pbio.1001765.s015.docx]

**Table S2:** Matched parameters (Mean ± standard deviation)

| **Averaged quantity** | **Measured** | **Tethered simulation** |
| --- | --- | --- |
| Virus speed | 14.3 ± 5.5µm*min^-1^ | 14.9 ± 0.3µm*min^-1^ |
| Distance between branches | 37.7 ± 30.1nm | 35.1 ± 25.4nm |
| Filament length | 121.1 ± 99.9nm | 113.3 ± 87.3nm |
| Number of attached filaments | 3.9 ± 1.5 | 4.1 ± 0.2 |
