# Supplementary material for: Electron Tomography and Simulation of Baculovirus Actin Comet Tails Support a Tethered Filament Model of Pathogen Propulsion
Source: PLoS Biol. 2014 Jan 14;12(1):e1001765. doi: 10.1371/journal.pbio.1001765 (PMC3891563; doi:10.1371/journal.pbio.1001765)
Supplement: Table S3 — Predicted parameters of the simulation assuming continuous tethering of filaments to the virus surface compared to experimentally observed parameters. (DOCX) [file pbio.1001765.s016.docx]

**Table S3:** Predicted and observed parameters (Mean ± standard deviation)

| **Averaged quantity** | **Measured** | **Tethered simulation** |
| --- | --- | --- |
| Angular deviation of paths | 17.4 ± 14.8 | 22.0 ± 24.4° |
| Number of branches/tail length | 121.8 ± 19.2µm^-1^ | 103 ± 0.8µm^-1^ |
| Filament length/branch | 168.7 ± 11.3nm | 119 ± 1.5nm |
| Number filaments/tail length | 189.5 ± 33.3µm^-1^ | 113 ± 0.8µm^-1^ |
| Number subsets/tail length | 13µm^-1^ | 10.6 ± 1.7µm^-1^ |
| Number filaments/subset | 14.2 ± 8.3 | 10.9 ± 2.17 |
| Filament length/tail length | 22.4 ± 2.6µm | 12.2 ± 0.1µm |
| Angle between filaments and virus trajectory | 50.9 ± 23.7° | 52.6 ± 26.8° |
