# Supplementary material for: Electron Tomography and Simulation of Baculovirus Actin Comet Tails Support a Tethered Filament Model of Pathogen Propulsion
Source: PLoS Biol. 2014 Jan 14;12(1):e1001765. doi: 10.1371/journal.pbio.1001765 (PMC3891563; doi:10.1371/journal.pbio.1001765)
Supplement: Text S1 — Details of mathematical simulation. (DOCX) [file pbio.1001765.s017.docx]

**Text S1**

**Mathematical modelling**

We have taken a quasi-static approach to model the key players engaged in actin-based propulsion. In this type of approach it is assumed that the system seeks a level of minimal energy whereby slow processes can be approximated by series of states infinitesimally close to equilibrium. Several forces are assumed to act on the virus, namely pushing and pulling forces exerted by the actin network, Brownian motion and the friction exerted by the cytosol. Calculation of the balance between all these energy potentials defines the new position of the virus. The simulation scheme is a time stepping procedure with fixed length $\Delta t$ (typically 0.9ms). Each time step has stochastic parts for updating the state and length of the actin filaments together with the Brownian forces arising from thermal fluctuations and a deterministic part for updating the position of the virus. These stochastic and deterministic calculations are iterated in an alternating sequence.

The mathematical model is described below in two parts. The first part deals with the stochastic behavior of the actin filament network and the second part with how the virus position is affected by the actin filaments, friction and Brownian motion.

**Actin filaments:** Actin filaments are modelled as rigid straight-line segments, immobile relative to the cytoplasm. Branched ends can be tethered to the virus or be detached from it. p78/83 is located on one end of the virus, acts as a nucleator and in the case of the “tethered” simulation it links the filaments to the virus surface potentially in cooperation with VASP. Possible anchoring points for tethers occur at different positions on the rear end of the virus, taken as a semicircle, and described by
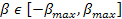
$\beta\epsilon\left[ -\beta_{max},\beta_{max} \right]$, the angle between the axis of the virus and the semicircle radius through the point on the virus surface. Detached barbed ends are capped stochastically. All stochastic processes occur with probabilities $\lambda\Delta t$ with rates $\lambda$, where the decision about the occurrence of an event is made using the integrated Matlab random number generator which gives evenly distributed random numbers between 0 and 1. The processes changing the states of actin filaments are:

- Polymerization at barbed ends (see Fig. 6A-C, 1,4): This is modelled deterministically by elongating filaments at attached or uncapped barbed ends by $v\Delta t$ per time step, where the polymerization speed $v$
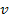
 of tethered filaments is given by $v=\left( v_{max}-v_{min} \right)\sin\varphi+v_{min}$
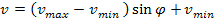
with maximal and minimal speeds $v_{max}$ and$v_{min}$, respectively and with the angle $\varphi$ between filament and the normal to the virus surface. For detached uncapped filaments ${v=v}_{max}$
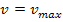
 is chosen. The angle dependent reduction of the polymerization speed models a dependence of the polymerization speed on the pushing force [1].
- Branching (see Fig. 6A-C, 3): Branching occurs at attached barbed ends with a fixed branching angle $\gamma_{b}$
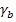
 (measured at 74°) and with a rate depending on the distribution of attached barbed ends. p78/83, a homolog of WASP [2] is capable of Arp2/3 activation. Regarding to our model this provides a limited capacity of branching and tethering new filaments, which might be locally reduced further as a consequence of crowding. This is modelled by assuming that each attached barbed end contributes to the inhibition of branching in its neighbourhood. The branching rate
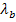
$\lambda_{b}$ is computed by reducing its maximal value
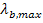
$\lambda_{b,max}$ by a factor depending on the total number of attached filaments and on the inhibition effect of attached filaments (with numbers
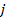
$j$) close to the mother filament with number
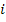
$i$:

${\lambda_{b}=\lambda_{b,max} \frac{N-n}{N}\left( 1-\sum_{j\neq i} \varrho\left( \frac{\beta_{i}-\beta_{j}}{\Delta\beta} \right) \right)}_{+}$
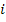


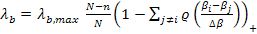


where $n$
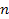
 is the number of attached barbed ends and
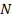
$N$ the maximal capacity of tethering barbed ends. The position of the barbed end of the $i^{th}$
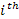
 filament along the virus surface is given in terms of the angle
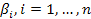
$\beta_{i}, i=1,\ldots,n$ . The function $\varrho$
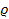
is a triangular hat function with height one and support of length 2. The parameter $\Delta\beta$
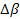
can be interpreted as an inhibition range of a barbed end. The subscript $+$ replaces possible negative values inside the parenthesis by zero. Branching always occurs on the side of the mother filament closer to the virus or with the same probability for both sides for the “tethered random branching” model (see Fig. 6I).

- Capping (see Fig. 6A-C, 5): Capping occurs at detached uncapped barbed ends with a rate
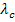
$\lambda_{c}$
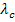
. It stops further polymerization.
- Depolymerisation at pointed ends (see Fig. 6A-C, 6): After capping and sufficient aging, depolymerisation is started at a randomly chosen time with rate $\lambda_{D}$
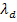
 and continues with fixed speed $v_{d}$
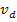
 thereafter. The age threshold is chosen to mimic the approximate length of the tail as observed experimentally.
- Nucleation of new filaments: At each time step, at most one new filament can be nucleated. Nucleation only occurs (with rate $\lambda_{n}$
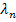
) if the number of tethered filaments falls below the threshold $n_{0}$
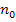
. The attachment point is chosen along the anchoring region according to the probability density


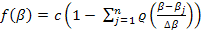
$f\left( \beta\right)=c\left( 1- \sum_{j=1}^{n} \varrho\left( \frac{\beta-\beta_{j}}{\Delta\beta} \right) \right)$

where the constant $c$
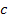
 is chosen to make $f$ a probability density. This has the effect of new filaments avoiding crowded regions along the virus surface. Immediate branching is assumed, which also determines the direction of the new mother filament and the new branch by assuming equal lengths initially.

**The virus:** The shape of the virus is described as a rectangle with length
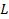
$L$ and width $2r$
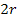
, with two semicircles of radius
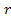
 $r$ attached. In each time step, a small translation (by at most
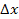
$\Delta x$) and rotation (by at most
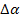
$\Delta\alpha$) of the virus is chosen randomly. This approximates random forcing by thermal fluctuation of the virus in the cytoplasm. The values of
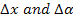
$\Delta x$and $\Delta\alpha$ correspond to a diffusivity of the virus, which is several orders of magnitude smaller than published data for purified baculovirus in TE buffer [3], but sufficient for sharpening the difference between models with and without tethering of filaments (see below). After the Brownian forcing step, the final position of the virus after the time step is determined by a quasi-stationary force balance, where friction of the virus against the cytoplasm, the steric interactions between filaments and the virus, and pulling forces on the virus by tethered filaments are considered. The force balance is realized by minimization of the potential energy


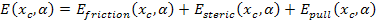
$E\left( x_{c},\alpha\right)=E_{friction}\left( x_{c},\alpha\right)+E_{steric}\left( x_{c},\alpha\right)+E_{pull}\left( x_{c},\alpha\right)$.

The position of the virus, being described as a two-dimensional rigid body, can be given in terms of its centre of gravity
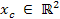
$x_{c} \in\mathbb{R}^{2}$ and an orientation angle $\alpha$.
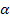
 More precisely, if $x$
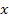
varies in the reference configuration
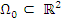
$\Omega_{0}\subset\mathbb{R}^{2}$ of the virus (corresponding to $x_{c}=\alpha=0$)
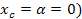
, then the configuration with centre of gravity
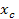
$x_{c}$ and orientation $\alpha$
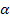
 contains the points $x_{c}+R\left( \alpha\right)$ with the rotation matrix

$R(\alpha)=\left( \begin{matrix} \cos\alpha& -\sin\alpha\\ \sin\alpha& \cos\alpha\end{matrix} \right)$
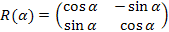
.

For given centre of gravity $x_{c}^{old}$
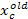
 and orientation $\alpha^{old}$
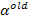
 at the old time step, the frictional contribution to the potential energy is given by

$E_{friction}\left( x_{c},\alpha\right)=\frac{\mu_{friction}}{2\Delta t}\int_{\Omega_{0}} \left| x_{c}+R(\alpha)x-x_{c}^{old}-R\left( \alpha^{old} \right)x \right|^{2}dx$
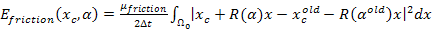
,

with the friction coefficient $\mu_{friction}$
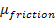
. With the identification


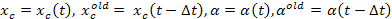
$x_{c}=x_{c}\left( t \right), x_{c}^{old}=x_{c}\left( t-\Delta t \right),\alpha=\alpha\left( t \right),\alpha^{old}=\alpha(t-\Delta t)$

and with small $\Delta t$
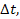


$\nabla_{x_{c}}E_{friction}\approx\dot{x}_{c}\mu_{friction}Area\left( \Omega_{0} \right) , \partial_{\alpha}E_{friction}\approx\dot{\alpha}\mu_{friction}\int_{\Omega_{0}} \left| x \right|^{2}dx$
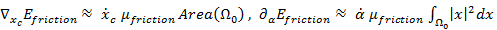

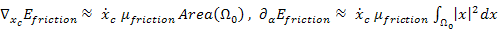


showing that our model corresponds to linear frictional resistance against translation and rotation of the virus. The steric interactions between the virus and the filaments would ideally be modelled by an interaction between rigid bodies using a side condition of non-penetration of the virus by filaments. Minimization under side conditions can be avoided, if the steric interaction is softened such that penetration of the virus by filaments is penalized by a contribution to the potential energy

$E_{steric}\left( x_{c},\alpha\right)=\mu_{steric}\sum_{j} \int_{0}^{L_{j}} d\left( x_{j}\left( s \right);x_{c},\alpha\right)ds$
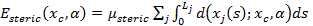
,

where the sum goes over the numbers $j$
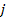
 of all filaments extending into the virus. By
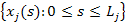
$\left\{ x_{j}(s):0\leq s\leq L_{j} \right\}$
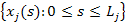
an arclength parameterization of the piece of the filament inside the virus is given. The function $d\left( x;x_{c},\alpha\right)$
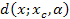
 is a measure of how far the point $x$
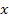
 lies inside the virus. It was chosen such that the integrals can be computed easily ($d=\hat{d}\left( 2r-\hat{d} \right)$ with the distance $\hat{d}(\leq r)$
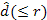
 between $x$
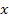
and the virus surface). The bigger the constant $\mu_{steric}$
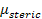
 is chosen, the smaller the violations of the steric constraint will be, but also the harder it is to solve the minimization problem. Our choice (see Table S1) seems to be a reasonable compromise.

Finally, pulling by both kinds of tether is modelled as a linear elastic spring force with a maximal threshold, above which the tether breaks. Thus, if the barbed ends of the tethered filaments in the “tethered” simulation or the branch points in the “tethered during branching” simulation are denoted by $x_{b,1},\ldots,x_{b,n}$
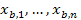
and the respective nearest points on the virus by $x_{v,1}\left( x_{c},\alpha\right),\ldots,x_{v,n}\left( x_{c},\alpha\right)$
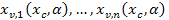
then the potential energy contribution from the tethers is given by

$$E_{pull}\left( x_{c},\alpha\right)=\sum_{j=1}^{n} E_{tether}\left( \left| x_{b,j}-x_{v,j}\left( x_{c},\alpha\right) \right| \right),$$

$$E_{tether}\left( z \right)=\left\{ \begin{aligned} \mu_{tether}\frac{z^{2}}{2}, z<z_{threshold}, \\ 0, else. \end{aligned} \right.$$

As soon as a tether breaks the corresponding tether point is designated as detached after the minimization step.

**Parameters and predictions:** The parameters of the model are listed in Table S1 together with a justification. Some parameters are known from the literature, some can be directly observed in electron tomograms (ET), and some have been used for fitting averaged quantities, extracted from light microscopy and ET. The success of the fitting procedure in the case of the “tethered” simulation is demonstrated in Table S2. After parameterization of the model, its predictive capacities can be tested by comparing further averaged quantities from simulation and ET (see Table S3). Filament tethering to the baculovirus rear proved essential in the presence of Brownian motion. We found that both the architecture of the actin tail, especially in the number of filaments per subset and the persistence of the virus tracks *in vivo* were best simulated assuming continuous filament tethering to the pathogen. The ratio of polymerization and branching rates needed to be matched to reproduce tail architecture and virus propulsion. The capping rate and depolymerisation rate did not affect the simulation stability. At too high nucleation rate a high number of subsets with too few filaments per subset were generated, whereas at too low values sharp turns were not corrected and the tracks tortuous. The branching rate relative to polymerization rate had to be properly matched. With too little branching relative to the speed movement became unstable with more turns and thinner tails, while with excess branching the tails became too thick. The model was insensitive to a broad range of values taken for the frictional coefficient. In general the forces of the spring and the friction and pushing forces had to be mutually tuned. For example if we assumed high friction with low force there was little or no movement. The maximal deflection distance of the springs was restricted to a range between 1nm and 10nm since smaller values reduced the life time of springs and longer values distorted the viral movement. In our simulation only the ratios between
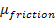
$\mu_{friction}$, $\mu_{steric}$ and
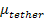
$\mu_{tether}$ affect the dynamics and can be determined by fitting to experiments. The absolute values have been chosen to demonstrate that the results can be interpreted in terms of reasonable values for the forces. The maximal force sustained by a tether is 1.9pN and the average friction force with a virus speed of 14.9µm*min^-1^ (Table S2) is 14.5pN. This is approximately equal to the total pushing force of the filaments, which is reasonable in view of a stall force of 5pN per filament [1] and an average number of 4 attached filaments (Table S2).

**Supporting references**

1. Mogilner, A., and Oster, G. (1996). Cell motility driven by actin polymerization. Biophys J *71*, 3030-3045.

2. Machesky LM, Insall RH, Volkman LE (2001) WASP homology sequences in baculoviruses. Trends Cell Biol 11: 286-287.

3. Toivola, J., Ojala, K., Michel, P.O., Vuento, M., and Oker-Blom, C. (2002). Properties of baculovirus particles displaying GFP analyzed by fluorescence correlation spectroscopy. Biol Chem *383*, 1941-1946.

4. Vialard JE, and Richardson CD. (1993) The 1,629-nucleotide open reading frame located downstream of the Autographa californica nuclear polyhedrosis virus polyhedrin gene encodes a nucleocapsid-associated phosphoprotein. J Virol. *67*, 5859-66.
